# Twenty-first century knowledge mapping on oral diseases and physical activity/exercise, trends, gaps, and future perspectives: a bibliometric review

**DOI:** 10.3389/fspor.2024.1410923

**Published:** 2024-08-07

**Authors:** Thamires Campos Gomes, José Lucas Gomes Moura, Daiane Claydes Baia-da-Silva, Rafael Rodrigues Lima, Patrícia de Almeida Rodrigues

**Affiliations:** ^1^ Graduate Program in Dentistry, Institute of Health Sciences, Federal University of Pará, Belém, Brazil; ^2^ Laboratory of Functional and Structural Biology, Institute of Biological Sciences, Federal University of Pará, Pará, Brazil

**Keywords:** oral health, stomatognathic system, sports medicine, exercise, athletic performance

## Abstract

Maintenance and improvement of an individual's overall well-being require a multidisciplinary approach that encompasses everything from oral health care to regular physical exercise. The notion that poor oral health can influence general health and athletic performance has sparked an interest in this relationship. This study offers an overview of relevant research and a knowledge map,and discusses publication metrics and key topics concerning the relationship between physical activity or exercise and oral diseases. We searched the Web of Science database for articles published in the 21st century that addressed the relationship between physical activity and oral diseases. Under the stipulated inclusion criteria, a rigorous selection process yielded 276 from 3,883 retrieved articles. The articles were classified by what was assessed as follows: occurrence of oral diseases in athletes or sports enthusiasts (*n* = 174); impact of physical activity or exercise on the oral cavity (*n* = 59); effects of oral changes on sports performance and physical fitness (*n* = 31); and the connection between oral health status, physical activity or exercise, and systemic conditions (*n* = 12). Orofacial trauma has received the most attention among all investigated oral diseases. However, there is a need for greater attention of dysfunctional habits that can contribute to premature tooth wear, as well as oral inflammatory diseases that can have systemic implications. This mapping can encourage the development of new primary research.

## 1 Introduction

Oral health plays an important role in maintaining overall health and well-being. Lack of proper oral health care can lead to the proliferation of various pathogens, resulting in oral diseases such as dental caries, periodontal disease, and subsequent tooth loss (1). The relationship between oral and general health has been extensively studied, particularly regarding the systemic release of chemical mediators resulting from the inflammatory burden generated by oral diseases, which may negatively affect preexisting inflammatory processes (2).

Recently, there has been a growing interest in the interplay between oral diseases and physical exercise. Extensive research has been conducted to highlight the protective role of physical exercise in mitigating the severity of oral diseases such as periodontal disease (3). Another line of investigation has explored the potential impact of oral diseases on athletic performance (4). This attention has grown due to a rising interest in adopting healthier lifestyles that include both dietary choices and physical activity.

Improving quality of life requires a multidisciplinary approach. Oral health is consistently associated with quality of life indicators in the general population. Engaging in physical exercise at higher-than-usual levels is associated with a 31% lower risk for infectious diseases (5).

Physical activity involves movements performed by skeletal muscles that result in energy expenditure. On the other hand, physical exercise is a set of planned, structured, and repetitive physical activities aimed at improving or maintaining physical fitness (6).

Physical exercise is associated with improved mental health, cognition, depression, anxiety, and neurodegenerative diseases (7). Regular physical activity is consistently correlated with an improved quality of life. Physical activity or inactivity is the primary environmental factor that modulates cardiorespiratory fitness, which is the body's ability to absorb and distribute oxygen to muscles and organs during prolonged physical exercise. Low cardiorespiratory fitness is strongly linked to a higher risk of cardiovascular diseases and stroke, and serves as an independent risk factor for type 2 diabetes (8).

Engaging in sports, at the amateur or professional level, is an excellent activity for regular physical exercise. The focus of most athletes is to surpass limits and break records. Therefore, understanding the biological mechanisms and factors that influence sports performance has attracted greater interest for evaluating and monitoring the oral health of athletes and physically active individuals. In addition to infectious and inflammatory diseases, orofacial and dental injuries have gained special attention due to their extended recovery time and potential irreversible damage to athletes. Managing orofacial injuries and developing preventive measures are at the forefront in sports dentistry.

It is essential to concisely evaluate the potential relationships between oral diseases or alterations and physical activity or exercise, as well as the role of oral diseases in athletic performance. For this purpose, an assessment of the main points already studied, and the gaps still present in the literature is necessary, to more precisely direct future research in the area. Therefore, the present study provides an overview of related research and a knowledge map and discusses publication metrics and the key topics regarding the relationship between physical activity/exercise and oral diseases.

## 2 Methods

### 2.1 Search strategy

An advanced search was conducted on the 29th March, 2024 in the Clarivate Analytics Web of Science Core Collection (WoS-CC) database, using a search strategy (Supplementary Table S1). The search strategy focused on terms like physical activity, exercise, sports, oral diseases, and oral disorders, with a specific limitation to publications involving humans from the 21st century. This study used bibliometric analysis tools described by Né et al. (9).

### 2.2 Study selection and data extraction of bibliometric parameters

Two independent reviewers (T.C.G and J.L.G.M) scrutinized titles and abstracts, to identified primary human studies that addressed the relationship between physical/exercise activity and oral disease, published in the 21st century. This includes cross-sectional studies, longitudinal studies, and randomized controlled trials were selected without language restrictions, without distinction by gender or age of participants, method of exercise or physical activity analysis, sport/exercise performed, athlete category, or exercise frequency. Publications that solely focused on exercises with therapeutic intentions, such as physiotherapy exercises, and studies that did not examine genuine oral diseases or alterations but simulated changes instead, were excluded, as well as conference proceedings, editorials, laboratory studies, reviews, letters, and conference abstracts. If uncertainties were present, the entire article was reviewed for a comprehensive evaluation. Any disagreements were resolved by a third reviewer (D.C.B-.S).

Following a meticulous article reading and selection process, TXT and EXCEL files were exported from the WoS-CC. The Excel file was used to extraction/analysis of bibliometric analysis, information such as the author, title, DOI, WoS-CC citation count, keywords, publications years, journal of publication, corresponding author addresses, and affiliations. The graphic representation of the origin of the articles visualized using MapChart (www.mapchart.net).

The text file was then imported into Visualization of Similarities Viewer software (CWTS, Leiden University, Leiden, Netherlands) and used to construct bibliometric networks of keywords.

### 2.3 Content analysis

After analyzing the publication metrics, the researchers created a data extraction spreadsheet in Excel to systematically retrieve pertinent information from each article. This included details such as type of study, sample number, gender, age group, method of analyzing exercise or physical activity (validated questionnaires, self-report or objective measures), sport/exercise performed, category of athletes (professional/amateur), exercise frequency (regular exercise routine of participants or exercise sessions specifically conducted for the study), oral disease/change, oral disease analysis method, blood, salivary, or gingival fluid parameters assessed, main objectives, and main results. This analysis was conducted by two independent reviewers (T.C.G and J.L.G.M), and any disagreements were resolved by a third reviewer (D.C.B.-S).

## 3 Results

### 3.1 Selected studies and bibliometric analysis

**Figure 1:**
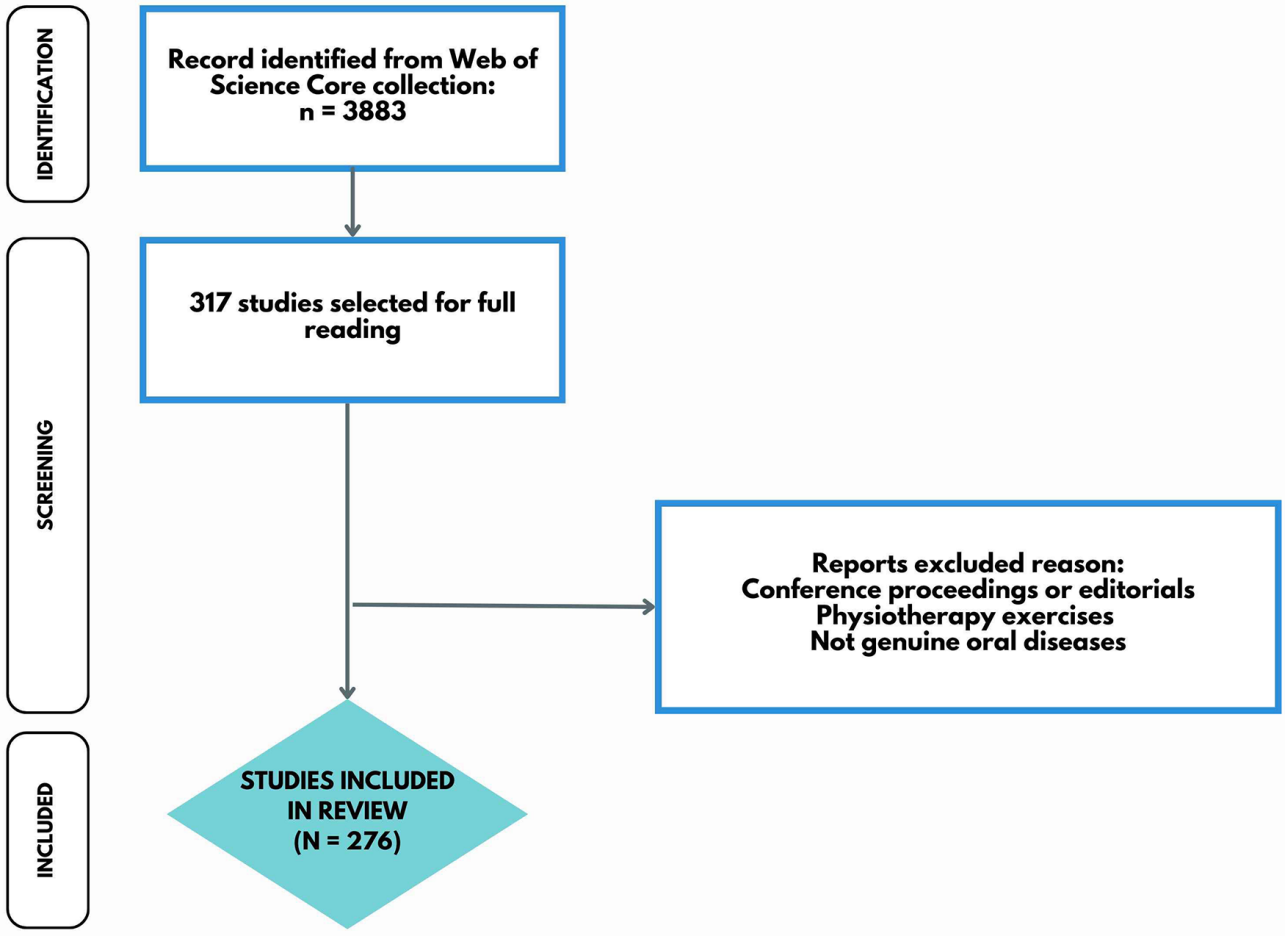
Flow diagram of screened publications.

The WoS-CC search yielded 3,883 publications, and their titles and abstracts were reviewed to identify those that fulfilled the inclusion and exclusion criteria. In total, 276 articles were selected (Figure 1). The complete list of publications encompassing the relationship between physical activity/exercise and oral diseases is provided in Supplementary Tables S2–S5.

**Figure 2:**
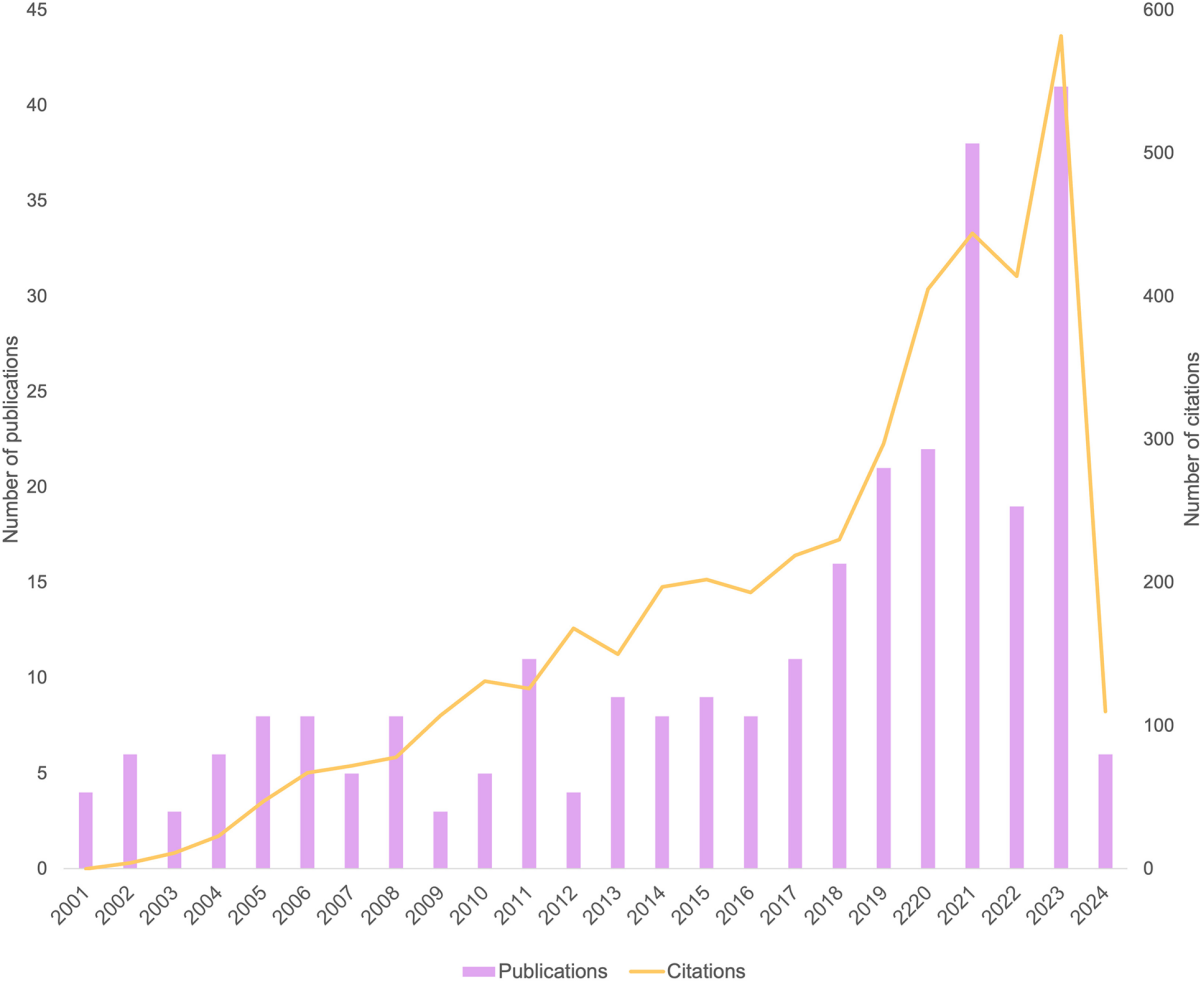
Number of publications and citations per year.

The Figure 2 depicts the trend in the number of publications over the years. A visual inspection of the data reveals a steady increase in the overall number of publications, with a particularly notable rise beginning in 2018. This trend may reflect a growing awareness and interest in this research area during that period.

#### 3.1.1 Most cited articles

The article that garnered the highest number of individual citations (500 citations in WoS-CC) was “Evaluate the effect of the five main causes of facial injuries on the severity of maxillofacial trauma.” The second and third articles with the highest citation counts within the Web of Science main collection also focused on orofacial or dental trauma; the titles of these articles are “Effect of mouthguards on dental injuries and concussions in college basketball” (138 citations in WoS-CC) and “Dental trauma and level of information: mouthguard use in different contact sports” (94 citations in WoS-CC), respectively.

#### 3.1.2 Contributing authors

A total of 1,250 authors, regardless of their position in the authorship order, contributed to advancing knowledge concerning the interplay between oral diseases and exercise/physical activity. The authors who made the most significant contributions were Filippi (10 articles, 244 WoS-CC citations), Mark (10 articles, 120 WoS-CC citations), Kuhl (6 articles, 93 WoS-CC citations), and Needleman (6 articles, 157 WoS-CC citations). The top 10 authors who published the most on the subject, along with their levels of contribution, are shown in Table 1.

**Table 1:** Top 10 most contributing authors.

Position	Authors	NP	PY start	FA	CA	LA	TC
1°	Marks, L	10	2012	1	0	9	1,020
2°	Filippi, A	10	2002	0	0	10	244
3°	Needleman, I	6	2013	1	2	3	157
4°	Kuhl, S	6	2011	0	6	0	93
5°	Kaschke, I	5	2010	0	5	0	72
6°	Fernandez, C	5	2015	3	1	1	65
7°	Pohl, Y	4	2002	0	4	0	151
8°	Ashley, P	4	2013	0	4	0	151
9°	Krastl, G	4	2008	0	4	0	105
10°	Haas, AN	4	2015	0	3	1	32

NP, number of publications; PY start, year of publication start; FA, first author; CA, co-author; LA, last author; TC, total of citations.

#### 3.1.3 Citation count by authors

Gassner, Tuli, Hachl, Rudisch, and Ulmer, H, had the most cited articles and shared the top position for most cited authors. In second position for authors with the highest number of citations were Filippi (244 WoS-CC citations) followed by Al-Zarani, Bissada and Borawski (161 WoS-CC citations).

**Figure 3:**
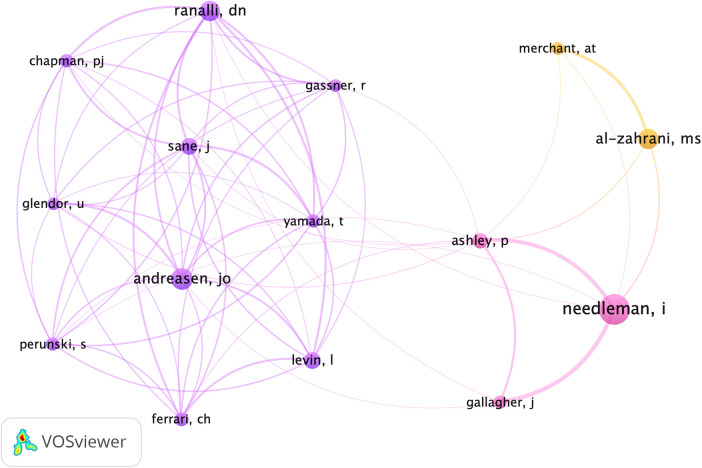
Representation of co-citations. The larger the circle, the more times the author was cited in the references of the 276 selected articles. The lines indicate the co-occurrence between citations, with thicker lines representing a higher level of interaction between them.

When examining the number of co-citations (Figure 3), which represents the frequency with which an author was cited in the references of the 276 selected articles, the author with the highest number of co-citations was Needleman with 76 citations. Needleman often collaborates with Ashley and Gallagher, distinguished members of the UCL Eastman Dental Institute in London, United Kingdom. The second most co-cited author was Andreasen (50 co-citations), a prominent figure in global dental trauma research.

#### 3.1.4 Journal rank list and impact factor (IF)

**Figure 4:**
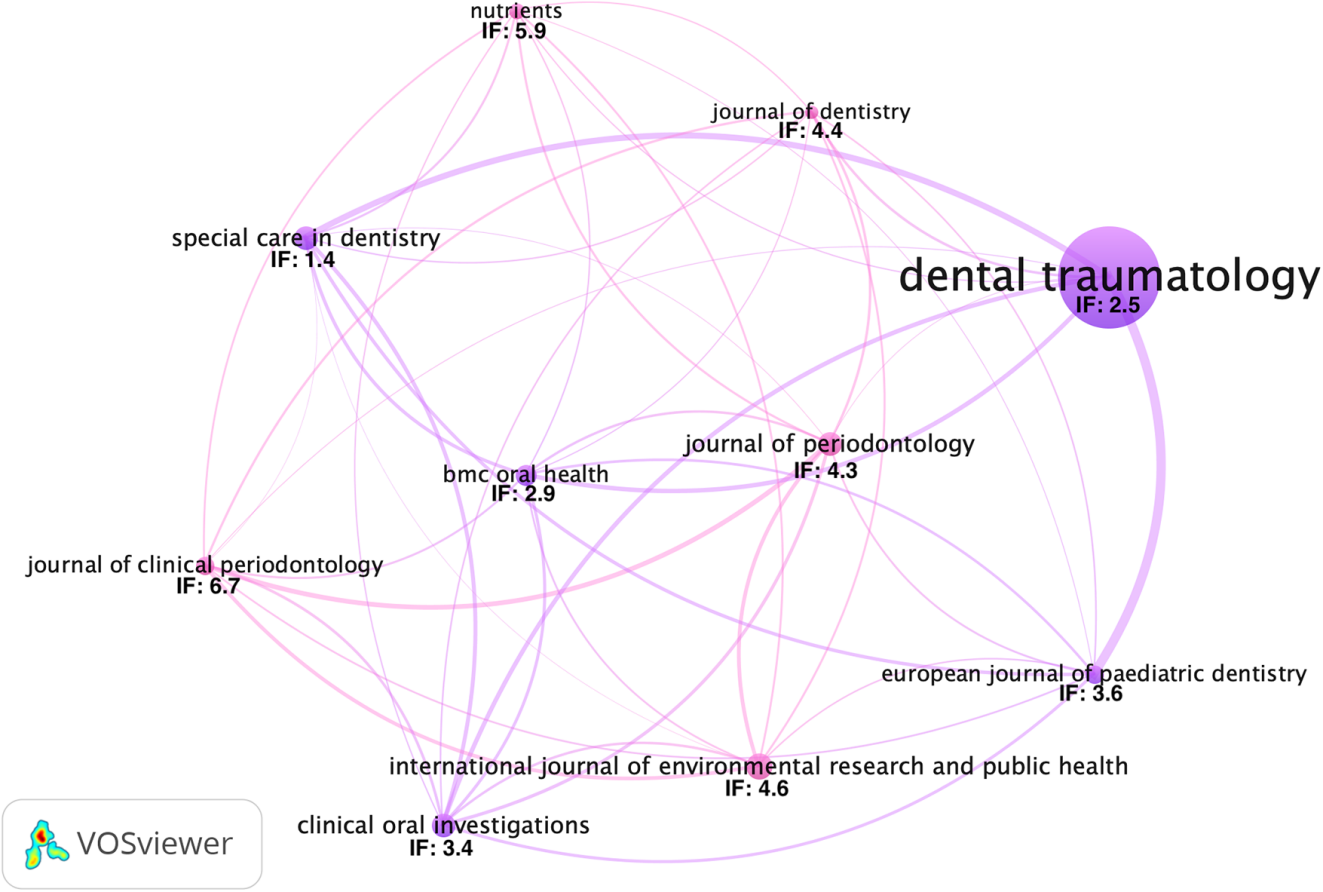
The 10 journals with the highest number of publications and their respective impact factors in 2022. The number of citations was used as a tiebreaker criterion. The lines indicate the co-citation between the journals, with thicker lines representing a higher level of interaction between them.

The evident trend for the growing interest in orofacial injuries is highlighted by the considerable number of publications in the journal *Dental Traumatology* (52 articles; IF: 2.5) (Figure 4). This prominence is notable among the 117 journals that disseminated the 276 selected articles on the relationship between physical activity and oral diseases.

Among the journals, those with the highest impact factors were the *British Journal of Sports Medicine* (three articles; IF: 18.6) and the *European Journal of Epidemiology* (one article; IF: 16.6). Of the 158 articles published in dental journals, notable impact factors were observed for the *Journal of Dental Research* (1 article; IF: 7.6), *Journal of Clinical Periodontology* (6 articles; IF: 6.7), and *International Endodontic Journal* (1 article; IF: 5.0).

#### 3.1.5 Geographical distribution

**Figure 5:**
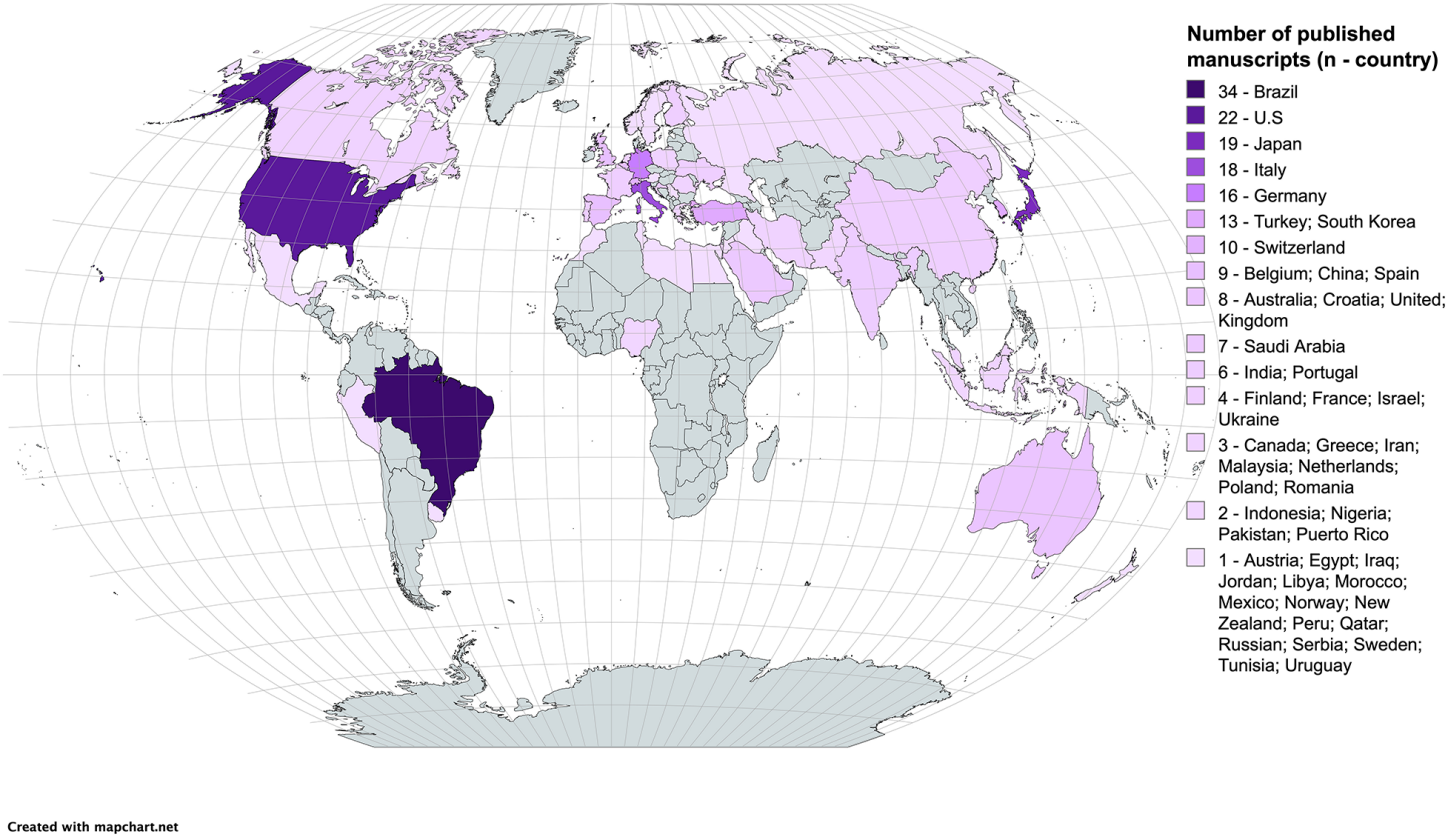
Global distribution of publications by country, based on corresponding authors.

A total of 49 countries contributed 276 publications based on the geographical distribution of the corresponding authors (Figure 4). Brazil had the highest number of publications (34 articles, 316 citations in WoS-CC), followed by the United States of America (22 articles, 496 citations in WoS-CC), Japan (19 articles, 344 citations in WoS-CC), and Italy (18 articles, 199 citations in WoS-CC) (Figure 5). English language accounted for 271 articles, while the remaining articles were in Portuguese (2), German (2), Russian and Korean (1).

#### 3.1.6 Keywords

**Figure 6:**
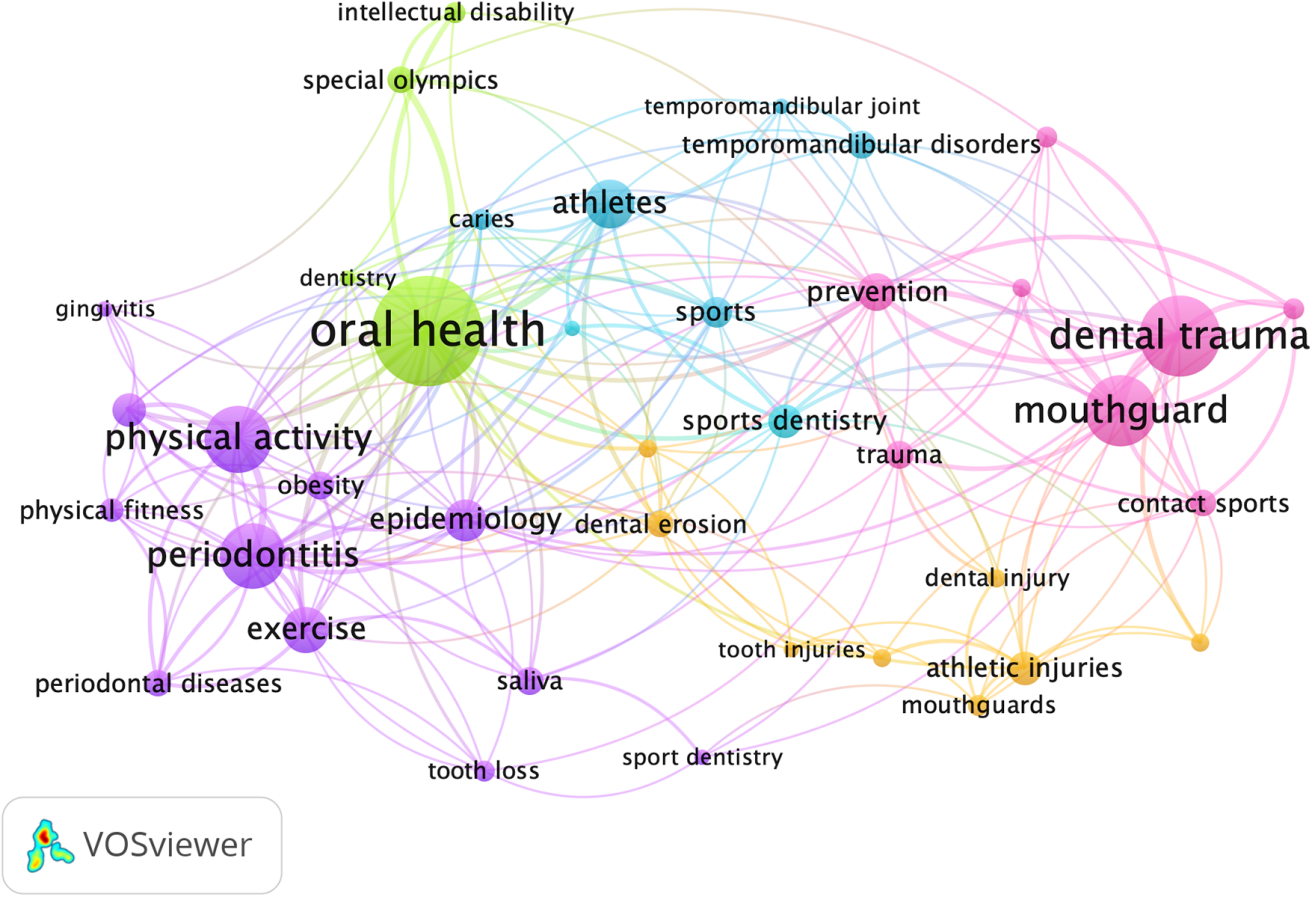
Keywords among authors. The lines indicate the co-occurrence between the keywords.

Of the 566 author keywords, 39 distinct words with at least five occurrences were identified (Figure 6). The most prevalent keywords included “oral health” (*n* = 50), “dental trauma” (*n* = 35), “mouthguard” (*n* = 30), “physical activity” (*n* = 28), “periodontitis” (*n* = 27) and “athletes” (*n* = 19).

### 3.2 Knowledge mapping

**Figure 7:**
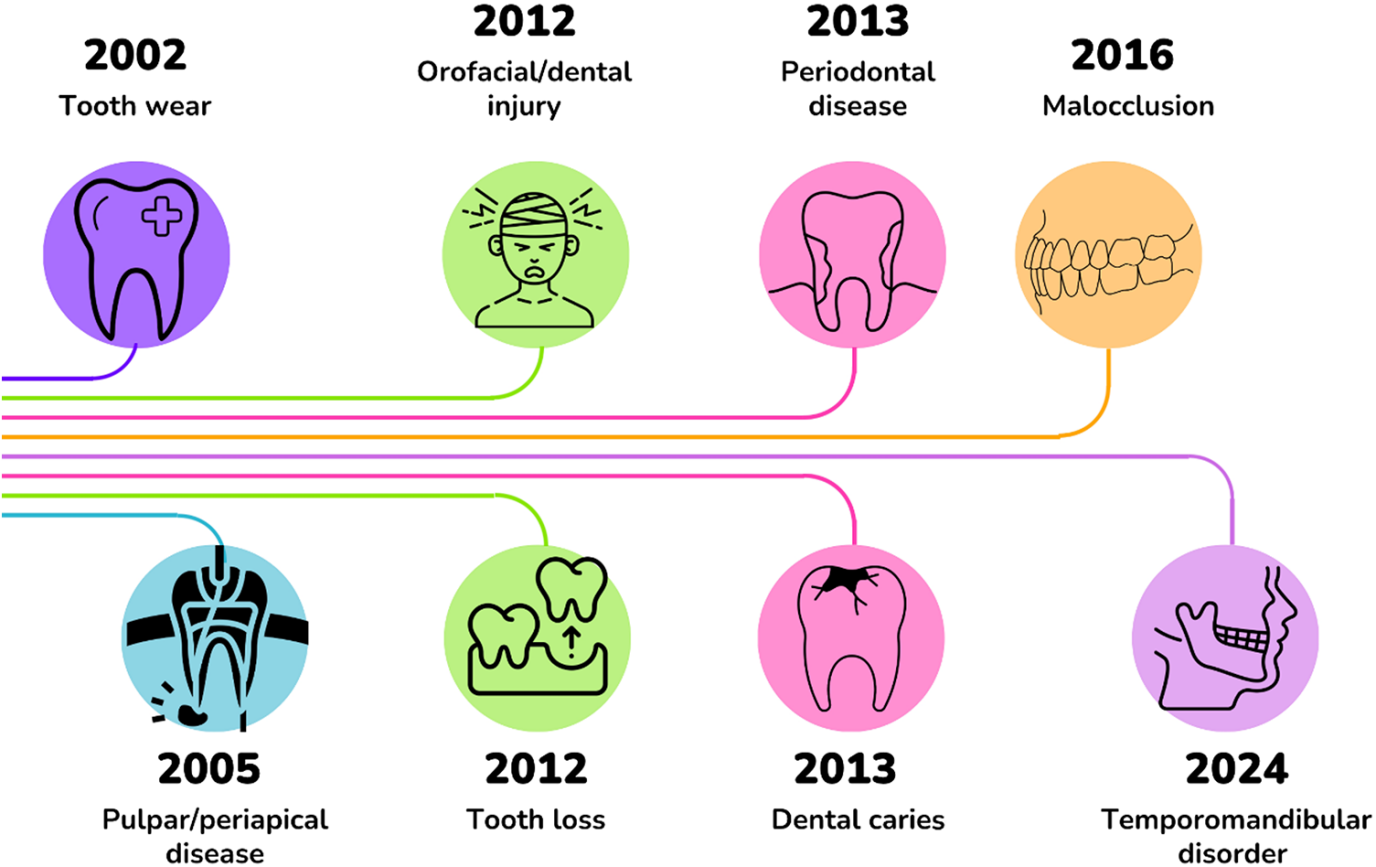
Timeline of publications according to the year with the highest density of publications for each oral disease.

The selected studies addressed various oral diseases and alterations. We assessed the research concentration on the major ones by calculating the density of the investigations. This was performed by dividing the number of publications for each oral disease in each year by the total number of publications in that year. The year with the highest publication density for each oral disease is presented in Figure 7.

However, when examining cumulative publications across the century, it was evident that orofacial injuries consistently remained the most prominent oral disease of interest among researchers, whereas pulpal and periapical diseases garnered comparatively less attention. Despite having the highest publication density in 2005, there has been an increase in the average number of publications per year, particularly over the last 7 year.

#### 3.2.1 Study design

**Figure 8:**
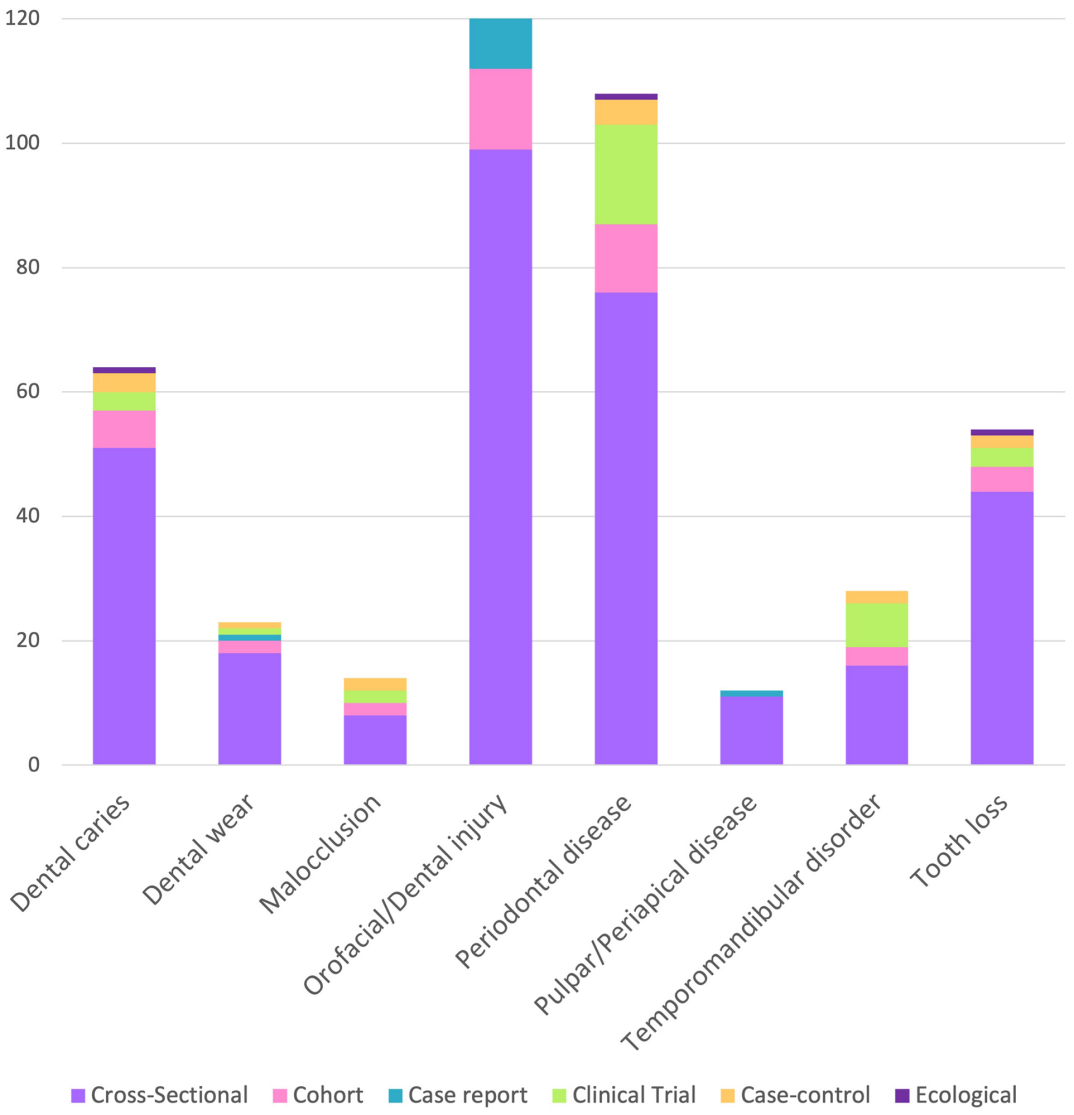
Distribution of oral disease evaluated by study design.

More than 70% of the publications (197 articles) were cross-sectional studies, followed by 30 clinical trials, 25 cohort studies, 12 case reports, 11 case-control studies, and one ecological study. The distribution of primary oral diseases based on the study design is depicted in Figure 8.

#### 3.2.2 Research field

**Figure 9:**
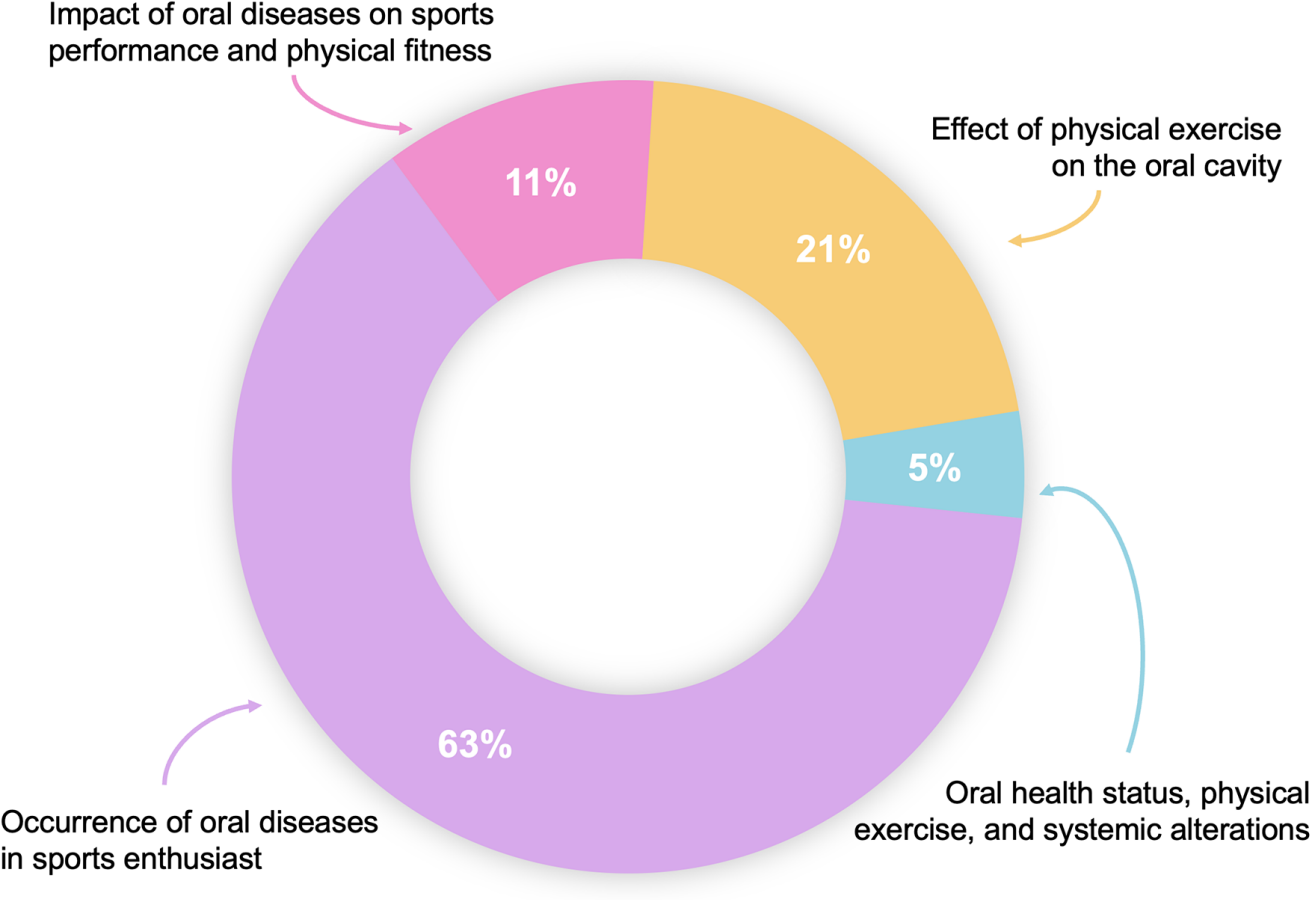
Article distributions by research fields.

In our analysis, we identified four primary research areas concerning the relationship between oral diseases and physical activity. These areas, ranked by the number of studies, include assessment of oral disease occurrences in athletes or sports enthusiasts, with or without treatment intervention (*n* = 174); effects of physical exercise on the oral cavity (*n* = 59); impact of oral changes on sports performance and physical fitness (*n* = 31); and relationship between oral health status, physical activity, and systemic conditions such as obesity, cardiovascular diseases, and diabetes (*n* = 12). This categorization reflects the predominant focus of the studies we reviewed, with a detailed distribution presented in Figure 9.

#### 3.2.3 Content analysis

The oral diseases most widely associated with physical exercise were orofacial and dental injuries, evaluated in 126 articles, followed by periodontal disease, dental caries, and tooth loss, evaluated in 108, 64, and 54 articles, respectively. To evaluate oral condition, 64 articles relied solely on self-reported that obtained the participant history, without any clinical assessment conducted by examiners. Evaluations of orofacial or dental injuries based solely on self-reported comprised 50%, whereas 42% were clinically assessed injuries. The remaining studies relied on injury reports from sports organizations. Dental caries and tooth loss were primarily assessed using the decayed, missing, and filled teeth index. In a small number of studies (*n* = 6), tooth loss was evaluated using self-reported participant history questionnaires.

To assess physical fitness, aerobic endurance exercises such as ergometric bicycle, treadmill, elliptical, and the Cooper's test were used. Strength exercises included isometric weightlifting, quadriceps isometry, and handgrip strength tests. Functional balance tests such as the timed up-and-go and standing balance tests were also performed.

Among the publications analyzed, 60% focused on researching professional and amateur athletes of different ages, while 2% studied individuals who engaged in sports sporadically. Sports, such as soccer, basketball, swimming, volleyball, and athletics, were the most frequently studied. They were evaluated for the presence of various oral diseases and conditions; orofacial and dental injuries, periodontal disease, caries, tooth loss, and dental erosion were the most common.

High rates of dental caries were observed in athletes. The average DMFT (Decayed, Missing, and Filled Teeth) index ranged from 1.87 to 7.71 among athletes from various sports disciplines. Among participants in the Special Olympics, the rate of untreated caries ranged from 21% to 61%.

The selected studies that assessed tooth loss can be divided into two main categories: studies with athletes and studies with older populations. Our bibliometric analysis revealed that a substantial number of studies focus on the relationship between physical activity and oral health, particularly tooth loss. For older populations, several studies highlighted a positive association between a greater number of teeth and higher levels of physical activity (10–12).

Only 12 articles evaluated pulpal diseases or apical periodontitis. Of these, nine are studies involving athletes, with only one being a case report and the rest assessing the prevalence of oral diseases in this population.

A total of 100 articles assessed injury occurrences in athletes. Evaluation of the knowledge levels among athletes, parents, and coaches regarding actions to be taken for sports traumas and the need for mouthguard use during sports activities were the primary focus of research with athletes, which aimed to prevent orofacial injuries.

Four articles used o pH and salivary flow to assess the risk factors for dental erosion, whereas salivary microbiota was studied to evaluate the risk of dental caries and periodontal disease in nine articles. The evaluations of salivary and gingival crevicular fluid biochemical parameters were conducted to examine markers of the immune system such as interleukin (IL)-6, IL-1β, IL-8, IL-10, tumor necrosis factor-alpha, and C-reactive protein.

## 4 Discussion

The main goal of this study was to provide information to elucidate the understanding of the knowledge-construction process surrounding oral diseases and physical activity or exercise. By using bibliometric analysis tools and mapping of the current century literature, it was possible to identify the most discussed topics, the least addressed or neglected points and discuss future perspectives for research on the relationship between oral diseases and physical exercise. Upon analyzing the data found, the literature indicated that the presence of oral diseases and alterations can negatively impact quality of life and physical fitness levels (13, 14). Moreover, regular physical exercise was beneficial not only for overall health but also for oral health (15, 16). However, contrary to the popular belief that athletes are generally healthier than the rest of the population, athletes had higher rates of oral disease (17–20). Current studies have indicated that participation in competitions is associated with an increased risk of infectious diseases. The pursuit of breaking records and constantly pushing limits may be linked to the dysregulation of an athlete's immune system (5).

The first mention in the literature of the relationship between physical exercise and oral changes concerned athletes, particularly in boxing, and the occurrence of orofacial injuries, and the development of devices to minimize damage (21). Over the years, dental research focusing on exercise has ceased to be exclusive to athletes and has begun to encompass the general population. At the beginning of this century, studies conducted with nonathletes followed a similar publication pattern to those conducted with athletes. Starting in 2013, we observed an increase in research involving the general population, which suggests a strong interest in studying the impact of oral diseases on physical fitness and quality of life of the general population, as well as regular non-competitive physical exercise practice.

The three articles with the highest number of citations among the selected articles investigated orofacial and dental injuries as well as the level of knowledge among sports practitioners regarding injury prevention. The occurrence of orofacial or dental injuries during sports has been a long-standing concern. Since 1890, there have been reports on the use of devices to minimize damage caused by trauma in this area (21). Orofacial and dental injuries have evident negative effects on athletes, justifying the historical interest of researchers in this subject, which is been reflected in the large number of scientific articles addressing the topic. Concerns regarding the level of knowledge among athletes and coaches about the prevention and first aid for these injuries have remained constant in this century (22–27).

The most productive author was Andreas Filippi, who contributed 11 articles as the last author. One of his main areas of research focus is interdisciplinary dental traumatology. He is the Head of the Department of Oral Surgery at the University Center for Dental Medicine Basel, Switzerland and has the highest number of contributions in this field. Other indicators of the prominence of oral and dental injuries over other oral alterations include the result that the journal with the highest number of publications in this area was *Dental Traumatology* and that Jens Ove Andreasen was the second author with the highest number of co-citations among the selected articles. He is recognized as the most productive author in the field of dental traumatology worldwide (28).

Brazil is the most productive country in the fields of publications related to oral diseases and physical exercise. It is the only country that officially recognizes sports dentistry as a dental specialty to date. This interest from the “country of soccer dates back to 1958, when dentist Dr. Mário Trigo accompanied the Brazilian national football team during the World Cup held in Sweden. Curiously, the inclusion of a dentist in the team staff, who was responsible for the athletes’ health, occurred simultaneously with the team's first world championship.

The study of how diseases affect populations, known as epidemiology, played a fundamental role in most of the selected articles. The second most frequent keyword among publications was “epidemiology” highlighting the epidemiological role of most selected articles. Many studies have evaluated the prevalence of major oral diseases such as orofacial lesions, caries, tooth loss, pulpal diseases, periodontal disease, and temporomandibular disorders.

Good general health practices, along with physical activity, have been associated with improved oral health practices and a lower occurrence of tooth decay in the general population (29–31). However, high rates of dental caries were observed in athletes. A high-risk profile for dental caries may be a consequence of a diet high in carbohydrates and a significant intake of sports and/or energy drinks with high sugar content (19, 20), proliferation of microorganisms and decrease in the level of S-IgA (32). Nevertheless, there are still few studies evaluating the risk factors athletes face for developing diseases such as tooth decay.

According to Tibúrcio-Machado et al., half of the world's adult population has at least one tooth with apical periodontitis. However (33), the present study found a low number from the articles that evaluated pulpal and periapical diseases. The difficulty determining diagnosis and the need for complementary evaluations, such as imaging examinations, may justify such occurrences. The majority of the studies selected in this review evaluated the epidemiological assessment for the need for endodontic treatment in athletes (34–36).

Among the oral diseases evaluated in this review, periodontal disease was the oral alteration with the highest number of clinical studies (16 articles). Many of these studies had observed the positive effects of physical exercise (37–40). Furthermore, the hypothesis that a high oral inflammatory burden may be associated with lower levels of physical fitness has led to an increasing number of studies examining the association between periodontal disease and physical fitness during the past decade (13, 41–45). The prolonged use of substances by athletes, such as anabolic androgenic steroids, has resulted in increased gingival inflammation (46).

The need for fluid and nutrient replenishment during physical activities, especially when performed at high intensity, encourages athletes to consume large quantities of energy and sports drinks (47). Due to the acidic pH of these beverages, the relationship between their consumption and prevalence of dental erosion has been widely studied. Studies have shown that a high intake of sports drinks can be considered a risk factor for dental erosion, although an isolated association cannot be statistically proven. Weekly training duration and low fluid intake during sports activities are associated with higher rates of dental erosion. Medeiros et al. (48) her important risk factor for dental erosion that should be evaluated among individuals engaged in physical activities. These factors have been associated with so-called “early dental aging,” a concern in contemporary dentistry that deserves special attention in sports practitioners.

The high prevalence of oral diseases in athletes raises important questions about the biological mechanisms of these diseases in this population. A more cariogenic microbiota has been observed in athletes compared to the general population, especially after sports practice (49–52). It is known that an imbalance in the oral microbiota, associated with frequent carbohydrate consumption or reduced saliva flow, can lead to caries, and excessive plaque accumulation increases the risk of periodontal diseases (53). Oral diseases such as periodontal disease and apical periodontitis have been associated with increased systemic inflammatory biomarkers and oxidative stress (54, 55). The findings of Mendoza-Nunez et al. (37) suggest that practicing Tai Chi has antioxidant and anti-inflammatory effects that are linked to the improvement of periodontal disease in the elderly. However, the mechanisms of the impact of oral diseases, especially inflammatory ones, on the sports performance of elite athletes still do not seem to be clear.

This study is limited by the use of a single database, WoS-CC, which, despite its comprehensive coverage, may not have retrieved papers published exclusively in other databases, potentially leading to different results and incomplete data. Additionally, the choice to include only publications from the 21st century excludes studies from earlier periods. Therefore, it is not possible to assert that all articles from this century addressing this topic were retrieved. Care must be taken when establishing trends and identifying gaps. The bibliometric methodology is not based on a qualitative analysis of articles; its main objective is to evaluate the behavior of the literature, such as the main terms used, productive authors, and journals, enabling the identification of key trends and gaps in scientific knowledge. This indicates that it does not make judgments regarding the quality of the studies.

Although the findings of this study do not directly enable informed decision-making regarding protocol choices and comprehensive clinical safety evaluations, they motivate the development of new primary research. Given the trend towards a more integrative approach in science, research that explores the relationship between dentistry and exercise has contributed to the reintegration of the oral cavity into the overall body system. To achieve this, it is crucial to focus on dysfunctional habits that can contribute to premature tooth wear as well as oral inflammatory diseases that can have systemic implications.

## 5 Gaps and future perspectives

The predominant oral condition of interest remains orofacial trauma, despite the growing number of studies demonstrating the high prevalence of oral diseases such as dental caries and periodontal disease. The current literature is still heavily focused on evaluating the prevalence of oral diseases; however, there is an urgent need for a deeper understanding of the factors that lead athletes to have high rates of oral diseases and for a more thorough investigation of the impact of these diseases on sports performance. To address these issues, we suggest that more studies, especially randomized clinical trials, be conducted.
