# Supplementary material for: Twenty-first century knowledge mapping on oral diseases and physical activity/exercise, trends, gaps, and future perspectives: a bibliometric review
Source: Front Sports Act Living. 2024 Aug 7;6:1410923. doi: 10.3389/fspor.2024.1410923 (PMC11335734; doi:10.3389/fspor.2024.1410923)
Supplement: Supplementary file 1 [file Datasheet1.pdf]

Supplementary Material

Supplementary Table 1. Web of Science Core Collection' search strategy

Manuscripts	Web of Science Core Collection' syntax
n = 3883	TS=(Exercise OR Exercises OR Fitness OR "Physical Exercise" OR "Physical Exercises" OR "Acute Exercise" OR "Acute Exercises" OR "Isometric Exercise" OR "Isometric Exercises" OR "Physical Fitness" OR "Aerobic Exercise" OR "Aerobic Exercises" OR "Exercise Training" OR "Exercise Trainings" OR "Resistance Exercise" OR "Exercise Physicians" OR "Physical Activity" OR "Physical Activities" OR "physical-exercise" OR "physical-exercises" OR "physical-fitness" OR "Physical performance" OR "physical-activity" OR "physical activity" OR "Physical Trainning" OR "Strength training" OR "aerobic fitness" OR "aerobic power" OR "cardiorespiratory fitness" OR "cardiovascular fitness" OR "exercise behavior" OR "exercise capacity" OR "exercise intervention" OR "exercise performance" OR "exercise physiology" OR "exercise program" OR "exercise referral scheme" OR "exercise screening" OR "exercise test" OR "exercise testing" OR "exercise therapy" OR "flexibility exercise" OR "functional exercise capacity" OR "Exercise habituation" OR "exercise habits" OR "functional fitness" OR "physical inactivity" OR "Athletic Performance" OR "Athletic Performances" OR "Sports Performance" OR "Sports Performances" OR "Athletic Injuries" OR "Sports Injury" OR "Sports Injuries" OR "Athletic Injury" OR Athletes OR Athlete OR Sports OR Sport OR Athletics OR Athletic OR "Muscle Recovery" OR "muscle exercise" OR "skeletal muscle exercise" OR "muscle exercises" OR "Muscle Activity")<br><br>AND<br><br>TS=("Stomatognathic Diseases" OR "Mandibular Diseases" OR "Maxillary Diseases" OR "Mouth Diseases" OR "Oral Manifestations" OR "Tooth Diseases" OR Bruxism OR "Dental Deposits" OR "Dental Leakage" OR "Dentin Sensitivity" OR Hypercementosis OR "Tooth Abnormalities" OR "Tooth Ankylosis" OR "Tooth Demineralization" OR "Tooth Discoloration" OR "Tooth Wear" OR "Tooth Resorption" OR Toothache OR "dental caries" OR " Root Caries" OR "Periapical Diseases" OR "Dental Pulp Diseases" OR "Focal Infection, Dental" OR "Periapical Periodontitis" OR "Periapical Abscess" OR Pulpitis OR "Dental Pulp Calcification" OR "Dental Pulp Necrosis" OR "Tooth, Nonvital" OR Malocclusion OR "Mouth, Edentulous"

	OR "Tooth Eruption, Ectopic" OR "Tooth, Impacted" OR "Tooth, Unerupted" OR "Tooth Injuries" OR "Tooth Loss" OR "Tooth Avulsion" OR "Tooth Fractures" OR "Cracked Tooth Syndrome" OR "Periodontal Diseases" OR "Periodontitis" OR "Aggressive Periodontitis" OR "Chronic Periodontitis" OR "Gingival Diseases" OR "Peri-Implantitis" OR "Periodontal Atrophy" OR "Periodontal Cyst" OR "Tooth Mobility" OR "Salivary Gland Diseases" OR Xerostomia OR "Diseases, Stomatognathic" OR "Stomatognathic Disease" OR "Mouth and Tooth Diseases" OR "Dental Diseases" OR "Dental Disease" OR "Disease, Dental" OR "Diseases, Dental" OR "Manifestation, Oral" OR "Manifestations, Oral" OR "Oral Manifestation" OR "Disease, Mouth" OR "Diseases, Mouth" OR "Mouth Disease" OR "Disease, Tooth" OR "Diseases, Tooth" OR "Tooth Disease" OR "Teeth Grinding Disorder" OR "Disorder, Teeth Grinding" OR "Grinding Disorder, Teeth" OR "Teeth Grinding Disorders" OR Bruxomania OR Toothaches OR Odontalgia OR Odontalgias OR "Caries, Dental " OR "Dental Cavity" OR "Dental Decay" OR "Dental Cavities" OR "Cavities, Dental" OR "Cavity, Dental" OR "Carious Lesions" OR "Carious Lesion" OR "Lesion, Carious" OR "Lesions, Carious" OR "Decay, Dental" OR "Carious Dentin" OR "Carious Dentins" OR "Dentin, Carious" OR "Dentins, Carious" OR "Dental White Spot" OR "Spot, Dental White" OR "Spots, Dental White" OR "White Spot, Dental" OR "White Spots, Dental" OR "Dental White Spots" OR "Disease, Periapical" OR "Diseases, Periapical" OR "Periapical Disease" OR "Pulp Diseases, Dental" OR "Diseases, Dental Pulp" OR "Pulp Disease, Dental" OR "Dental Pulp Disease" OR "Disease, Dental Pulp" OR Pulpitides OR "Inflammation, Endodontic" OR "Endodontic Inflammation" OR "Endodontic Inflammations" OR "Inflammations, Endodontic" OR "Injuries, Teeth" OR "Injury, Teeth" OR "Teeth Injury" OR "Injuries, Tooth" OR "Injury, Tooth" OR "Tooth Injury" OR "Teeth Injuries" OR "Disease, Periodontal" OR "Diseases, Periodontal" OR "Periodontal Disease" OR Periodontitides OR Pericementitis OR Pericementitides OR "Disease, Gingival" OR "Diseases, Gingival" OR "Gingival Disease" OR Epulides OR Epulis OR Gingivosis OR Gingivoses OR "Disease, Salivary Gland" OR "Diseases, Salivary Gland" OR "Gland Disease, Salivary" OR "Gland Diseases, Salivary" OR "Salivary Gland Disease" OR Xerostomias OR Hyposalivation OR Hyposalivations OR Asialia OR Asialias OR "Mouth Dryness" OR "Dryness, Mouth" OR “Oral Health” OR "temporomandibular disorders" OR "oral hygiene" OR "oral parafunctions" OR "orofacial injuries" OR "orofacial pain" OR "temporomandibular joint disorders" OR Stomatitis OR Mucositis. OR “Tooth Erosion“ OR “Dental trauma”)
