# Supplementary material for: Twenty-first century knowledge mapping on oral diseases and physical activity/exercise, trends, gaps, and future perspectives: a bibliometric review
Source: Front Sports Act Living. 2024 Aug 7;6:1410923. doi: 10.3389/fspor.2024.1410923 (PMC11335734; doi:10.3389/fspor.2024.1410923)
Supplement: Supplementary file 2 [file Datasheet2.pdf]

Supplementary Material

Supplementary Table 2. Index of studies examining oral disease occurrences in athletes or sports enthusiasts, with or without therapeutic interventions

Author (year)	*Main Objective*	*DOI*
Abdelrahman, et al. (2023)	Determine the status of erosive tooth wear and its related risk factors among competitive swimmers compared to non-swimmers	10.1007/s00784-023-05367-7
Abdullah, et al. (2015)	Investigate the level of knowledge among rugby players regarding first aid measures for dental injuries.	10.1111/edt.12191
Aljohani, et al. (2017)	Investigate the incidence of dental and facial injuries amongst taekwondo athletes in Saudi Arabia and their awareness regarding their prevention and management.	10.15537/smj.2017.11.21111
Alrowdan, et al. (2023)	Evaluate the severity and frequency of TMDs among weightlifters in fitness gyms in Saudi Arabia	10.7759/cureus.49113
Alves, et al. (2017)	To evaluate the level of knowledge, attitudes and habits of soccer and basketball athletes about the interrelationship between oral health and sport.	10.1590/1517-869220172305170315
Amy (2005)	To assesse the occurrence of orofacial injuries in the Central American and Caribbean Sports Games of the Puerto Rican Delegation over the past 20 years	10.1111/j.1600-9657.2005.00283.x
Andrade, et al. (2010)	Investigate the occurrence of dental trauma in athletes representing 42 participating countries in the Pan-American Games	10.1111/j.1600-9657.2010.00884.x
Andrade, et al. (2012)	Investigate the occurrence of oral trauma in athletes representing 25 participating countries in the Parapan American Games	10.1111/j.1600-9657.2012.01174.x
Antunes, et al. (2017)	To assess dental erosion prevalence, risk factors, and the potential link between isotonic drink consumption and increased dental erosion in amateur racing athletes.	10.2334/josnusd.16-0611

Aral, et al. (2017)	Compare the periodontal condition and gene expression levels of interleukin-1β, apoptosis-associated speck-like protein containing C-terminal caspase-recruitment domain, and caspase 1 in bodybuilders with those of a control group to evaluate their periodontal status.	10.2334/josnusd.16-0367
Aslan, et al. (2023)	Evaluate the incidence of temporomandibular disorders in athletes practicing defensive sports and their symptoms	https://www.nobelmedicus.com/tr/article/774
Ayadi, et al. (2023)	To investigate the effects of using fixed orthodontic appliance on dynamic balance, auditory/visual reaction times, and pain perception in elite athletes.	10.1080/08990220.2023.2197995
Azadani, et al. (2023)	Provide estimates of dental injuries, the mechanism of injuries and the utilization of mouthguards in high school sports.	10.1111/edt.12800
Azodo, et al. (2011)	Investigate the occurrence of orofacial injuries among basketball players and assess athletes' awareness of mouthguard usage.	10.1111/j.1875-595X.2011.00009.x
Bazina, et al. (2020)	To evaluate knowledge and attitudes of water polo coaches regarding dental trauma, dental emergency procedures and awareness about prevention of such injuries.	10.1111/edt.12551
Beachy (2004)	Investigate the occurrence, type, and severity of dental injuries in interscholastic athletes	PMID: 15592602
Bechor, et al. (2008)	Report of a Case of Dental Luxation in an Amateur Boxer	10.1111/j.1600-9657.2007.00545.x
Bergman, et al. (2017)	Investigate the occurrence of orofacial trauma and mouthguard use in professional handball players.	10.1111/edt.12323
Biagi, et al. (2010)	To assess athletes' awareness of sports as a risk factor for dental injuries	https://pubmed.ncbi.nlm.nih.gov/20635839/

Bissar, et al. (2010)	Investigate oral health in adolescents participating in the 2008 SO Germany.	10.1111/j.1365-263X.2010.01065.x
Bonotto, et al. (2016)	Acess the occurrence of TMJ in high-performance martial arts fighters and compare it with the prevalence among recreational athletes and non-athletes.	10.1111/edt.12238
Botelho, et al. (2021)	To evaluate the prevalence of periodontitis in professional footballers and its association with nutritional parameters and self-report non-traumatic injuries.	10.3390/nu13061792
Braham, et al. (2004)	Investigate the incidence of head, neck, and orofacial injuries in Australian Rules football athletes who did not use helmets	10.1016/S1440-2440(04)80263-9
Brancher, et al. (2021)	To investigate the association of the salivary pH and parameters of oral health in Brazilian para-athletes.	10.1111/scd.12589
Bruggesser, S, et al. (2020)	Measure the prevalence of traumatic injuries in judo with a particular focus on dental and lip injuries. A further aim was to assess the influences of increased overjet and wearing fixed orthodontic appliances.	10.1111/edt.12547
Buck, et al. (2021)	Report a long-term follow-up after the immediate treatment of multiple dental injuries.	10.24873/j.rpemd.2021.03.821
Butera, et al. (2022)	To evaluate the efficacy of two different oral hygiene protocols, that a hydroxyapatite-based toothpaste plus mouthwash or toothpaste alone.	10.3390/jcm11164893
Caglar, et al. (2005)	Investigate the occurrence of dental trauma in young and adult ice hockey players and assess their level of knowledge and usage of mouthguards	10.1111/j.1600-9657.2004.00271.x
Cetinbas, et al. (2008)	Determine the distribution and etiology of coronal fractures in permanent anterior teeth in children and evaluate the association with sports participation.	10.1111/j.1600-9657.2008.00647.x
Cohenca, et al. (2007)	Investigate the occurrence and severity of dental trauma by sport among athletes participating in intercollegiate sports	10.14219/jada.archive.2007.0326

Correa, et al. (2010)	To investigate dental injuries among soccer players, assess the knowledge level of team medical departments regarding mouthguards, and evaluate the protocols for managing dental trauma.	10.1590/S1678-77572010000600007
Costantinides, et al. (2023)	Identify the incidence and etiology of dental trauma associated with facial fractures over a 10-year period.	10.1111/edt.12835
Crincoli, et al. (2022)	To investigate the prevalence of temporomandibular disorders (TMJ) in competitive athletes in contact sports compared to a non-athletes.	10.3390/dj10100180
D'Ercole, et al. (2016)	To assess the oral health status of young competitive and non-competitive swimmers, including an evaluation of salivary cariogenic bacteria and the concentration of secretory IgA (S-IgA).	10.1590/1678-775720150324
Dagon, et al. (2019)	To evaluate the prevalence and severity of dental trauma among SO athletes with intellectual disabilities.	10.1111/scd.12343
de la Parte, et al. (2021)	To assess the oral health status and oral health habits of elite athletes according to the performed sports type.	10.3390/su13137282
de Sant'Anna, et al. (2004)	Investigate the DMFT and the salivary profile in female soccer players	https://www.minervamedica.it/en/journals/medicina-dello-sport/article.php?cod=R26Y2013N01A0071
de Souza, et al. (2011)	Investigate the presence of malocclusions in athletes aged 13 to 20 years from São Paulo Football Club.	10.1590/S1806-83242011000100004
de Souza, et al. (2020)	To evaluate the dental conditions, habits, and oral health care of volleyball athletes, and their knowledge of the importance of oral health in sports performance.	10.1590/1517-869220202603214217
de Souza, et al. (2021)	Investigate the occurrence and patterns of orofacial injuries in CrossFit practitioners.	10.1111/edt.12625

dello Diago, et al. (2021)	Report a case of traumatic loss of teeth in the esthetic area in the growing patient	10.3390/dj9010006
Dursun, et al. (2015)	Investigate the occurrence of dental trauma and knowledge of traumatic dental injuries among "weekend warriors" in Ankara, Turkey.	10.2334/josnusd.57.191
Duymus, et al. (2009)	Investigate the occurrence of orofacial injuries in university athletes.	10.1111/j.1600-9657.2009.00769.x
El Ouali, et al. (2023)	Determine the effect of malocclusion on maximal aerobic capacity in young athletes.	10.3390/sports11030071
Elareibi, et al. (2023)	To assess the knowledge and attitude of contact sports coaches regarding dental trauma and its prevention and management.	10.1002/hsr2.977
Emerich, et al. (2013)	Investigate the frequency of dental injuries during training and competitions, the habit of using mouthguards, and the level of awareness about prevention and first aid after dental avulsion.	10.1016/j.jsams.2012.10.002
Eroje, et al. (2020)	to determine the awareness of sports teachers on the occurrence and prevention of oral-facial trauma in southern Saudi Arabia.	10.4103/njcp.njcp_466_19
Esmaeilpoor, et al. (2021)	To evaluate the self-reported experience, knowledge of management, related factors, and prevention of orofacial injuries in Karate and Taekwondo athletes	10.1186/s13102-021-00363-4
Fagundes, et al. (2014)	Report two cases of dental trauma caused by a collision and a sports accident.	10.1111/edt.12117
Fakhruddin, et al. (2007)	Investigate the frequency of mouthguard use and the occurrence of dental and caries injuries in schoolchildren in Ontario	PMID: 17672954
Farhadian, et al. (2020)	Investigate the identifying factors associated with sports-related dental injuries.	10.1186/s13102-020-00217-5

Fasciglione, et al. (2007)	Comparison of dento-facial injuries, mouthguard usage, and knowledge about dental trauma among squash athletes from Switzerland and Germany	10.1111/j.1600-9657.2005.00415.x
Fernandez, et al. (2015)	Compare the dental treatment needs of participants in the 2013 and 2008 SO.	10.1186/s12903-015-0157-9
Fernandez, et al. (2015)	Investigate the occurrence of dental trauma among SO athletes in European and Eurasian countries.	10.1007/s00784-015-1403-x
Fernandez, et al. (2016)	To assess the oral health status and treatment requirements of young athletes with intellectual disability from 53 countries across Europe and Eurasia who took part in the SO European Games held in Antwerp.	PMID: 26949233
Ferrari, et al. (2002)	Investigate the occurrence of dental trauma in different sports modalities and assess the level of knowledge and use of mouthguards	10.1034/j.1600-9657.2002.00017.x
Ferruzi, et al. (2021)	To evaluate the impact of socio-demographic/socio-economic factors, oral hygiene habits and clinical oral health on the Oral Health-Related Quality of Life (OHRQoL) on athletes with disabilities.	10.14393/BJ-v37n0a2021-53664
Figueira, et al. (2020)	Determine the prevalence of oral diseases, relate to the consumption of sports and energy drinks and the use of mouthguards; to assess coaches' knowledge about the cause of orofacial trauma in athletes.	10.24873/j.rpemd.2020.11.712
Frese, et al. (2018)	To assess the management and prevention of dental caries and the effect of stannous fluoride products in athletes.	10.1038/s41598-018-34777-x
Frontera, et al. (2011)	Investigate the history of orofacial trauma in basketball players, and relate it to the use of mouthguards, facial types, mouth breathing presence, player position in the game, and assess the athletes' level of knowledge about trauma and mouthguards.	10.1111/j.1600-9657.2009.00781.x

Gadjiyev (2020)	To evaluate the effectiveness of a complex of therapeutic and preventive measures for symptomatic diseases in professional athletes.	10.26724/2079-8334-2020-4-74-24-28
Galic, et al. (2018)	To assess the occurrence of sports-related dental injuries in young athletes and compare the frequency of such injuries between high-risk and medium-risk sports. Additionally, evaluate athletes' attitudes and habits regarding mouthguard use.	10.1111/edt.12394
Gallagher, et al. (2019)	To explore athlete-reported oral health behaviours.	10.1038/s41415-019-0617-8
Gass, et al. (2016)	Investigate the frequency of dental trauma, the usage of protective gear including helmets, back protectors, and mouthguards, as well as the knowledge of primary care following dental trauma.	10.1111/edt.12242
Gassner, et al. (2003)	To evaluate the effect of the five main causes of facial injuries on the severity of maxillofacial trauma	10.1016/S1010-5182(02)00168-3
Gay-Escoda, et al. (2011)	Investigate the oral health status of professional football players from Barcelona FC and its relationship with the occurrence of sports injuries.	10.4317/medoral.16.e436
Gialain, et al. (2017)	This report presents a case of an injury caused by a punch like blow to the face during a handball college team practice session	10.1111/edt.12343
Gowdar, et al. (2023)	Evaluate the knowledge of school teachers regarding the management of orofacial injuries in Alkharj, Saudi Arabia	10.4103/jpbs.jpbs_609_22
Gray, et al. (2019)	Describe the oral health status of athletes with intellectual disabilities competing in the SO, GB National Games	10.1038/s41415-019-0049-5
Guinot, et al. (2021)	To investigate awareness and frequency of use of mouthguards in children and adolescents who engage in high-risk sports.	10.23804/ejpd.2021.22.04.2

Hacquin, et al. (2021)	To determine the prevalence of orofacial injuries and the proportion of handball players using a mouthguard in Eastern France.	10.1111/edt.12688
Hajiyev (2021)	Identify the occurrence of major dental diseases among professional athletes and the efficacy of biologically neutral drugs in their treatment.	10.26724/2079-8334-2021-4-78-27-31
Hajiyev (2022)	Identify the level of dental morbidity of professional athletes with different directions of the training process.	10.26724/2079-8334-2022-4-82-36-40
Halabchi, et al. (2007)	Investigate injury rates in female Shotokan karate athletes and propose possible predisposing factors	PMID: 24198704
Hersberger, et al. (2012)	Investigate the frequency of dental and facial injuries in water polo, athletes' habits regarding mouthguard usage, and their level of knowledge about emergency procedures after dental trauma.	10.1111/j.1600-9657.2011.01083.x
Horswill, et al. (2006)	Acess the influence of liquid consumption during exercise on the body's defense against enamel erosion: salivary flow rate and salivary pH.	10.1055/s-2005-865779
Hwang, et al. (2019)	Investigate the occurrence of facial trauma in handball players in South Korea	10.1097/SCS.0000000000005198
Ilia, et al. (2014)	Investigate the occurrence of oral trauma and the importance of using mouthguards in amateur rugby players.	10.1111/adj.12223
Ineichen, et al. (2021)	To assess the occurrence of dental trauma in professional alpine skiing and to determine its association with a skier's performance level, discipline, and/or years of skiing experience.	10.1111/edt.12643
James, et al. (2018)	Examine the changing patterns of incidence and causes of dental injuries in Canadian patients aged 0-18 years.	10.1017/cem.2017.52
Johnson, et al. (2023)	Evaluate the prevalence, distribution, and pattern of orofacial injuries among Kabaddi players from Delhi and the NCR region	10.1016/j.injury.2023.02.053

Kanemitsu, et al. (2023)	Assess the frequency and status of head injuries caused by teeth (HICBT) occurring under the supervision of schools in Japan	10.1111/edt.12839
Karp, et al. (2006)	Report a case of dental avulsion in a 9-year-old boy associated with a sports-related traumatic event	PMID: 16805362
Kasum, et al. (2023)	Evaluate the knowledge, attitudes, and practices of soccer players regarding traumatic dental injuries and the use of mouthguards	10.1111/edt.12862
Kececi, et al. (2005)	Investigate the occurrence and type of dental injuries in elite athletes from volleyball, taekwondo, and handball. Additionally, assess the athletes' level of knowledge regarding the use of mouthguards	10.1111/j.1600-9657.2004.00302.x
Khan, et al. (2022)	To assess sports and energy drink consumption, oral health status and impacts on daily activities and sports performance of elite athletes.	10.3390/nu14235089
Knight, et al. (2019)	Dental screening of all individuals registered with the Qatar Olympic Committee	10.1136/bjsports-2017-098770
Kragt, et al. (2019)	To assess oral health on the Olympic Games.	10.1080/00913847.2018.1546105
Labella, et al. (2002)	Investigate the occurrence of orofacial traumas in college basketball players, according to the use or non-use of mouthguards	10.1097/00005768-200201000-00007
Lang, et al. (2002)	Investigate the occurrence of dental injuries and assess the level of knowledge among handball athletes regarding the use of mouthguards	10.1034/j.1600-9657.2002.00123.x
Leroy, et al. (2012)	To evaluate the oral condition and dental treatment needs of participants in the SO Special Smiles program organized in Belgium in 2008.	10.1922/CDH_2704Leroy06
Lesic, et al. (2011)	Investigate the occurrence, type, and severity of orofacial injuries during basketball and the frequency of mouthguard usage.	PMID: 21755701

Lesic, et al. (2023)	Measure condylar movements and symphyseal movements in athletes with a history of orofacial injuries and compare them with uninjured athletes	10.3390/dj11080195
Liang, et al. (2023)	Investigate the distribution of different oral injuries within each injury mechanism and assess which mechanisms are more likely to lead to dental injury	10.1111/edt.12894
Lieger, et al. (2006)	Investigate the occurrence of orofacial and brain injuries in different sports modalities and assess the level of knowledge among athletes and officials regarding the use of mouthguards	10.1111/j.1600-9657.2006.00328.x
Ma (2008)	Investigate the occurrence of orofacial injuries, particularly dental injuries, in basketball and assess the athletes' knowledge of mouthguard usage during training and competitions.	10.1111/j.1600-9657.2008.00586.x
Macedo, et al. (2019)	Determine the prevalence of injuries and disorders of the stomatognathic system and associated factors in practitioners of Brazilian jiu-jitsu and investigate the use of mouthguards.	10.1038/s41598-019-44598-1
Maladiere, et al. (2001)	Investigate the demographic patterns of patients, fractures site, and causes of sports injuries.	10.1054/ijom.2001.0059
Margaritis, et al. (2011)	To assess the relative risks of tooth erosion, balancing various potential erosion covariates, using epidemiological methods	10.1016/j.jdent.2011.02.007
Marks, et al. (2018)	Identify the oral health status and treatment needs of SO athletes with intellectual disabilities	10.1007/s00784-017-2258-0
Marro, et al. (2019)	Determine the presence and severity of erosive tooth wear in athletes with intellectual disable who participated in the SO Belgium 2016.	10.1186/s12903-019-0727-3
Marshall, et al. (2005)	Investigate the effectiveness of protective equipment use by rugby athletes in preventing injuries	10.1093/ije/dyh346

Matei, et al. (2023)	Explore the correlation between salivary biomarkers of oxidative stress and their association with oral health in children engaged in competitive sports	10.3390/healthcare11222927
Mathew, et al. (2002)	Investigate the incidence of dental erosion in college athletes and assess its association with sports drink consumption	10.1159/000063927
Maxen, et al. (2011)	Investigate the occurrence of ocular and orofacial injuries in Floorball.	10.1111/j.1600-9657.2010.00960.x
Medeiros, et al. (2020)	To determine the prevalence of NCCLs in footballers and to address potential risk indicators.	10.1186/s12903-020-01200-9
Merle, et al. (2022)	Explore interactions between signs of periodontal inflammation and systemic parameters in athletes.	10.3390/jcm11175161
Merle, et al. (2022)	To evaluate oral health status and oral health behavior in young athletes including the comparison of competitive and amateur sports.	10.1111/sms.14143
Merle, et al. (2022)	Compare clinical oral conditions as well as the self-reported oral health status of biathletes and cross-country skiers to non-athletic controls.	10.1080/15438627.2022.2090251
Meshramkar, et al. (2007)	Report a case of severe dental erosion in a yoga practitioner.	10.1111/j.1875-595x.2007.tb00123.x
Mihalik, et al. (2005)	Reporting a case of multiple orofacial traumas associated with football	PMID: 15970958
Minty, et al. (2018)	To evaluate the oral health of elite rugby players compared to the general population, including an assessment of their gingival status and analysis of the microbiological composition and pH of their saliva.	10.1016/j.jdent.2018.10.001
Mueller, et al. (2001)	Investigate the occurrence of injuries in Little League Baseball players	10.3810/psm.2001.07.874

Muhtarogullari, et al. (2004)	To evaluate and treat TMJ in basketball players with a history of head and jaw sports injuries. Additionally, investigate the periodontal status	10.1111/j.1600-9657.2004.00267.x
Muller, et al. (2008)	Investigate the frequency of dental injuries in mountain bike athletes and assess the habits of helmet and mouthguard usage, as well as the level of knowledge about dental rescue kits.	10.1111/j.1600-9657.2008.00660.x
Musu, et al. (2021)	Report a case of multi-modular imaging approach to followed-up the sequelae of a sport-related traumatic injury.	10.3390/dj9030027
Nagappan, et al. (2019)	To assess the oral health status among sports university students in India.	10.4103/jpbs.JPBS_287_18
Nijakowski, et al. (2020)	To determine risk factors for erosive lesions in young sports professionals	10.3390/ijerph17093002
Nogami, et al. (2020)	Survey and evaluate the characteristics of mandibular fractures, and dental injuries that occurred during the practice of baseball and softball in Sendai, Japan.	10.1111/edt.12512
Novrinda,et al. (2023)	To identify factors associated with mouthguard use and construct a model based on these factors among basketball players in Indonesia	10.1186/s12903-023-03480-3
Onyeaso, CO (2004)	To assess the occurrence of orofacial traumas in athletes, according to the use of mouthguards, and assess the level of knowledge.	PMID: 14977285
Opazo-Garcia, et al. (2021)	To determine the prevalence of the most common oral pathologies in high-performance athletes during the emergency dental care performed at the Lima 2019 Pan American Games	10.1038/s41405-021-00078-1
Ostensjo, et al. (2017)	Determine the prevalence of painful TMJ in an adolescent population, identify potential predisposing factors and comorbidities	10.1155/2017/2164825

Othman, et al. (2023)	To assess the incidence of sports-related dental injuries and oral health status among Malaysian para-athletes.	10.1111/scd.12858
Owattanapanich, et al. (2023)	To define patient demographics, clinical and injury data, and outcomes after hockey-related injuries presenting to a US trauma centers	10.1177/00031348221136577
Ozcelik, et al. (2006)	To evaluate the effects of anabolic androgenic steroid abuse on gingival tissues in a group of bodybuilders and weightlifters	10.1902/jop.2006.050389
Padilha, et al. (2021)	Investigate the prevalence of orofacial trauma according to mouthguard use among a sample of Brazilian rugby union players.	10.1111/edt.12592
Papakosta, et al. (2008)	Investigate the specificities of orofacial injuries sustained during soccer practice.	10.1111/j.1600-9657.2007.00536.x
Perheentupa, et al. (2001)	Investigate the occurrence of dental injuries and the associated risk factors throughout life	10.1034/j.1600-9657.2001.170103.x
Persic, et al. (2006)	Comparison of dento-facial injuries, mouthguard usage, and knowledge about dental trauma among squash athletes from Switzerland, Germany, and France.	10.1111/j.1600-9657.2006.00379.x
Pezzememti, et al. (2005)	Investigate the oral health status of SO athletes and the influence of socioeconomic conditions	10.14219/jada.archive.2005.0291
Pinho, et al. (2023)	To assess the oral health condition and treatment needs of Special Olympics (SO) athletes in Brazil.	10.1111/scd.12836
Popa, et al. (2023)	Compare the differences in oral health between young athletes and children not involved in competitive sports	10.3390/children10060946
Pradhan (2018)	To determine the association between dental caries and BMI among SO athletes.	10.1111/jppi.12255

Pradhan, et al. (2019)	explore associations between oral and auditory health in SO athletes.	10.1111/scd.12406
Pradhan, et al. (2021)	To describe and evaluate the oral health status and dental treatment needs of children with intellectual disabilities attending SO from 2010 to 2018.	PMID: 33875048
Pullishery, et al. (2020)	To assess the periodontal status in 35–44-year-old bodybuilders in the Kingdom of Saudi Arabia.	10.4103/AIHB.AIHB_58_20
Queiroz, et al. (2021)	To evaluate the oral health status of a group of Brazilian rowers and to identify the main risk factors influencing their oral health.	10.1590/1517-869220212706202 1_0131
Rattai, et al. (2018)	To evaluate the rates of ice hockey-related oral injuries, duration of time lost due to oral injuries, and mechanisms of oral injuries in the province of Alberta over a 15-year period.	10.1111/edt.12387
Razzak, et al. (2019)	Examine the rate of mouthguard adoption in grappling sports within the United Kingdom, aiming to determine if participants in these sports are prone to dental injuries.	10.1038/s41415-019-0942-y
Rojas, et al. (2016)	To assess the oral condition and dental treatment needs of SO athletes from Poland, Romania and Slovenia	10.1111/idj.12205
Rojas, et al. (2021)	To evaluate the oral condition and treatment needs of SO athletes from Greece, Italy and Spain.	10.1111/scd.12557
Safaraliev (2020)	Increasing the level of dental care for professional athletes on the background of intense physical and psycho-emotional stress with the use of biologically neutral medicine.	10.26724/2079-8334-2020-3-73-106-111
Santos, et al. (2006)	Report a case of orofacial trauma involving a professional basketball player	10.1111/j.1600-9657.2006.00344.x
Schildknecht, et al. (2012)	Investigate the frequency of dental injuries among Swiss rugby league players.	10.1111/j.1600-9657.2012.01115.x

Schulte, et al. (2011)	Investigate the occurrence of dental caries in athletes with intellectual disabilities who participated in the SO Germany Summer Games.	10.1055/s-0030-1254170
Seifert, et al. (2014)	Investigate the occurrence, type, and severity of orofacial injuries during basketball and the frequency of mouthguard usage.	PMID: 25536987
Shah, et al. (2020)	To evaluate perception and awareness of dental injury in sports children and their coaches during contact sports.	10.7860/JCDR/2020/45055.14331
Shaharuddin, IM, et al. (2021)	Determine DMFT index and its relation to the quality of life among national contact sports athletes.	10.4103/JIOH.JIOH_162_21
Shimizu, et al. (2023)	To assess the mechanisms of injuries that leads to prevention by performing a qualitative analysis of the occurrence of dental trauma.	10.1111/edt.12808
Silva, et al. (2021)	To investigate if the consumption of acidic food and beverages, including energy drinks is associated with dental erosion in athletes.	10.1016/j.scispo.2020.12.004
Sirimaharaj, et al. (2002)	Investigate the patterns of consumption of acidic foods and beverages among various sports groups and examine any relationship between consumption patterns and dental erosion	10.1111/j.1834-7819.2002.tb00334.x
Spinas, et al. (2018)	Develop a clinical therapy protocol for a group of 20 athletes who have suffered traumas affecting hard dental and periodontal tissues.	10.23804/ejpd.2018.19.03.4
Stanbouly, et al. (2021)	Determine the occurrence of dentofacial injuries and concussions among athletes and their perceptions of mouthguards and their role in injury prevention.	10.1097/SCS.0000000000007615
Stojanac, et al. (2013)	Report a case of dental trauma during a soccer match.	10.1111/j.1600-9657.2012.01154.x
Storrer, et al. (2021)	To investigate if recurrent manifestation of oral herpes lesions is associated with other factors and impacts the OHRQoL in para-athletes.	10.1111/scd.12616

Tinoco, et al. (2021)	To evaluate the prevalence of sports-related dentofacial injuries, mouthguard use and attitudes regarding tooth rescue among cross-country mountain biking and field hockey athletes	10.1111/edt.12636
Tiryaki, et al. (2017)	Investigate the occurrence of dental injuries among basketball players and assess coaches and athletes' awareness of mouthguard usage.	10.23736/S0022-4707.17.06790-1
Tiwari, et al. (2014)	To assess the association between the rate and type of orofacial trauma during sports activities and the level of knowledge and use of mouthguards among professional athletes.	10.2334/josnusd.56.239
Tozoglu, et al. (2006)	Investigate the rate of craniofacial injuries in amateur football and understand the nature of these injuries	10.1097/01.scs.0000234982.54121.6b
Trihandini, et al. (2013)	To assess the dental treatment needs of SO Special Smiles athletes in Indonesia between 2004 and 2009.	10.1111/ipd.12010
Tulunoglu, et al. (2006)	Investigate the occurrence of dental injuries and assess the level of knowledge and usage of mouthguards among boxers and taekwondo athletes	10.1111/j.1600-9657.2006.00386.x
Udayamalee, et al. (2023)	Evaluate the use of mouthguards in a lower-middle-income country like Sri Lanka, guiding the planning of preventive strategies	10.1111/edt.12916
Vidovic, et al. (2015)	To evaluate the occurrence of dental and facial injuries, the habit of using mouthguards, and the awareness of injury prevention and first aid after dental avulsion among young taekwondo athletes in Croatia.	PMID: 26147815
Vinereanu, et al. (2022)	To evaluate the oral health status and treatment needs of intellectually challenged athletes taking part in SO	10.3390/healthcare10010140
Vougiouklakis, et al. (2008)	To evaluate the data on dental care provision at the Athens Olympic Village in 2004 during the Olympic and Paralympic Games.	10.1055/s-2008-1038489

Warnavin, et al. (2021)	To evaluate if Androgenic anabolic steroids influence periodontal clinical and microbiological parameters, and to assess the expression of total protein and interleukin-1β in gingival crevicular fluid.	10.1007/s00784-020-03679-6
Yang, et al. (2011)	Document the dental services provided at the Olympic Games.	10.1136/bjsm.2010.075283
Young, et al. (2015)	Review the literature and report 3 cases of sports-related dental injuries	10.1177/1941738113486077
Zadik, et al. (2008)	To evaluate the impact of distributing free boil-and-bite mouthguards to amateur athletes on the occurrence of dental injuries during sports activities, comparing their usage or not	10.7205/MILMED.173.12.1185
Zadik, et al. (2009)	Investigate the occurrence, etiology, and consequences of orofacial injuries among elite commando fighters of the Israeli Defense Force.	10.1111/j.1600-9657.2008.00708.x
Zakirulla, et al. (2021)	Analyze data from the records of the past five years of Saudi children seen in the dental trauma emergency.	Available Online at: www.jrmds.in
Zamora-Olave, et al. (2018)	To determine the occurrence of orofacial injuries in water polo players and assess the frequency and impact of mouthguard use on oral functions.	10.1111/edt.12434
