# Supplementary material for: Twenty-first century knowledge mapping on oral diseases and physical activity/exercise, trends, gaps, and future perspectives: a bibliometric review
Source: Front Sports Act Living. 2024 Aug 7;6:1410923. doi: 10.3389/fspor.2024.1410923 (PMC11335734; doi:10.3389/fspor.2024.1410923)
Supplement: Supplementary file 3 [file Datasheet3.pdf]

Supplementary Material

Supplementary Table 3. Index of studies that evaluated the effect of physical activity on the oral cavity

Author (year)	***Main Objective***	***DOI***
Al-Zahrani, et al. (2005)	To evaluate the association between an increase in the number of healthy behaviors and the occurrence of periodontal diseases	10.1902/jop.2005.76.8.1362
Al-Zahrani, et al. (2005)	Investigate the association between physical activity and periodontitis	10.1016/j.jdent.2005.01.004
Almohamad, et al. (2022)	The purpose of this study is to evaluate the association between physical activity, sedentary behaviour, and periodontal disease.	10.1111/jcpe.13669
Alves-Costa,et al. (2023)	Analyze the relationship between periodontitis and behavioral and metabolic risks in Brazilian adults over three decades	10.1007/s00784-023-05384-6
Anderson, et al. (2018)	To evaluate the cross-sectional association between physical activity and serum IgG antibodies against selected periodontal microorganisms.	10.1002/JPER.17-0709
Baumeister,et al. (2023)	Examine the effect of self-reported and accelerometer-assessed physical activity on the risk of periodontitis	10.1007/s00784-023-05109-9
Bawadi, et al. (2011)	To evaluate the relationship between physical activity, healthy eating habits, and periodontal health status.	10.1111/j.1600-0765.2010.01314.x
Bryant, et al. (2011)	Identify risk factors for dental caries and erosion in elite triathletes.	10.1055/s-0031-1277192
Chen, et al. (2022)	Investigate the relationship between denture-wearing and physical activity in older adults with different cognitive function states.	10.3389/fnins.2022.925398
Chen,et al. (2024)	Assess the potential relationship between physical activity, anxiety, and oral health conditions among university students in China	10.1016/j.heliyon.2024.e24529

Cho, et al. (2020)	Investigate the association between general physical activity level and TMD pain in Koreans in a large-scale national database established through a nationwide survey.	10.1111/cdoe.12519
Chun,et al. (2024)	Investigate the influence of physical activity on disabling temporomandibular disorder pain	10.1186/s13005-024-00407-3
D'Ercole, et al. (2013)	To assess the oral health status of young players and investigate the impact of physical exercise on the key factors that characterize the oral ecosystem.	Medicina dello sport 66, 1, S. 71-80, Lit.
Dantony,et al. (2024)	Evaluate the effects of adding aerobic exercise on pain threshold in participants with myogenous TMD and generalized pain sensitization	10.3390/app14051799
dos Anjos, et al (2023)	Evaluate the association between each domain of physical activity and oral health conditions in Brazilian adults	10.1590/1807-3107bor-2023.vol37.0071
Ericsson, et al. (2016)	To assess the association between sociodemographic factors, lifestyle and beneficial health behaviors, and oral/periodontal health among adolescents.	10.3109/00016357.2015.1112424
Figueroa, et al. (2020)	This study evaluated the efficacy of aerobic physical activity in managing musculoskeletal orofacial pain in individuals with TMJ compared to treatment with an occlusal stabilization appliance.	10.22592/ode2020n36a3
Frese, et al. (2015)	To evaluate the impact of resistance training on the occurrence of dental erosion, caries, and salivary parameters.	10.1111/sms.12266
Han, et al. (2013)	To evaluate the association between salivary Nitric oxide levels and periodontal disease, considering variables such as sociodemographic status, general and oral health, and health-related behaviors.	10.1902/jop.2012.120237
Han, et al. (2017)	To assess the relationship between oral health behavior and periodontal disease, considering sociodemographic and lifestyle variables, using a multivariate logistic regression analysis model.	10.1097/MD.0000000000006176

Han, et al. (2019)	To evaluate the relationship between regular walking and periodontitis in a Korean representative sample of adults according to socioeconomic status.	10.1038/s41598-019-49505-2
Huttunen, et al. (2023)	Investigate the associations between self-reported physical activity and measured physical fitness and oral health of young men.	10.1007/s10266-022-00717-5
Hwang, et al. (2022)	Investigate the association between healthy lifestyle (diet quality, physical activity, normal weight) and periodontal diseases in adults.	10.3390/ijerph19073871
Iwasaki, et al. (2018)	To explore the association between lifestyle, measured as the combination of four healthy lifestyle factors, and the development of new periodontitis or its progression and tooth loss risks in older adults.	10.1111/jcpe.12920
Iwasaki, et al. (2023)	Determine whether physical activity is associated with periodontitis in the Japanese population.	10.1111/jre.13095
Jang,et al. (2023)	Evaluate the relationship between strength training, oral health, and quality of life	10.3390/healthcare11162250
Jouhar, et al. (2021)	To evaluate an association of body mass index (BMI), diet, physical activity, and oral hygiene practices with decayed, missing, and filled teeth (DMFT)	10.3390/nu13010224
Kito, et al. (2019)	To test the combined program of textured foods with oral and physical exercises on the improvement of oral and physical function in older adult	10.1007/s12603-019-1216-8
Leal, et al. (2023)	Investigate the association between sleep disorders and probable sleep bruxism in children eight to ten years of age.	10.1111/odi.13917
Marruganti, et al. (2022)	To evaluate the association between adherence to Mediterranean diet and physical activity level with the periodontal status	10.1002/JPER.21-0643
Marruganti, et al. (2023)	To evaluate the association between lifestyle behaviors and clinical periodontal outcomes following Steps 1/2 of periodontal therapy.	10.1111/jcpe.13813
Marruganti, et al. (2023)	To evaluate the association between leisure-time/occupational physical activity and periodontitis.	10.1111/jcpe.13766

Mendoza-Nunez, et al. (2014)	Investigate the effect of Tai Chi on biological markers of oxidative stress in saliva and its relationship with periodontal disease in older adults.	10.1155/2014/603853
Merchant, et al. (2003)	To assess the association between physical activity and periodontitis	10.1023/A:1025622815579
Miettinen, et al. (2021)	To evaluate the association of TMD symptoms with physical fitness, physical activity and BMI among Finnish conscripts.	10.3390/ijerph18063032
Min, et al. (2023)	To evaluate the association between the consumption of milk and having severe periodontitis.	10.3390/nu15040914
Moleirinho-Alves, et al. (2021)	To assess the effects of three exercise programs on pain intensity, neuromuscular activation, and bite force of masticatory muscles in patients with TMD.	10.3390/jpm11111170
Moleirinho-Alves, PMM, et al. (2021)	To assess the effects of three exercise programs on the frequency, intensity, and impact of headaches in patients with TMD.	10.1590/1678-7757-2021-0059
Moleirinho-Alves, et al. (2021)	To assess the effects of aerobic exercise on pain, anxiety and OHRQoL in patients with TMD.	10.1111/joor.13239
Munoz-Torres, et al. (2014)	To evaluate the association between measures of adiposity and periodontitis in older adults.	10.1111/cdoe.12069
Munther (2019)	To determine the effects of both cigarette smoking and exercise on total salivary antioxidants and their impact on periodontal health status.	10.1016/j.sdentj.2018.09.002
Nasri-Heir, et al. (2019)	To evaluate the effect of nonstrenuous aerobic exercise on chronic masticatory myalgia patients and healthy controls by means of mechanical temporal summation and response to mechanical stimulation performed on the dominant forearm.	10.11607/ofph.2342
Omori, et al. (2018)	Examine the effect of exercise habits on periodontal diseases and metabolic pathology.	10.2147/TCRM.S153397
Pacheco, et al. (2022)	To evaluate the salivary Proteome, Inflammatory, and NETosis Biomarkers in Older Adult Practitioners or not of Physical Exercise	10.1155/2022/3725056

Park, et al. (2018)	Compare the effects of the oral health program and the walking exercise program on health outcomes for pregnant women	10.4040/jkan.2018.48.5.506
Park, et al. (2015)	Investigate if short-term weight control affects periodontal indices and serum and gingival crevicular fluid biomarkers in young Koreans.	10.1186/s12903-015-0094-7
Petrini, et al. (2018)	To evaluate the oral health status in children and to correlate it with time spent in physical activity per week.	10.23804/ejpd.2018.19.04.2
Pu,et al. (2023)	Investigate the association between different types and intensities of physical activities and periodontitis in a nationally representative sample of adults in the United States	10.1002/JPER.23-0070
Sadiq, et al. (2021)	To understand impact of physical activity on pain perception in patients presenting with acute pulpitis for endodontic therapy.	10.53350/pjmhs211582073
Samnieng, et al. (2013)	Analyse the relationship between health practices, oral health behaviors, and oral health status in the elderly.	10.1111/j.1741-2358.2012.00672.x
Sanchez, et al. (2020)	To assess the association between levels of physical activity and oral health in adults residing in Spain.	10.1038/s41415-020-1306-3
Sanders, et al. (2009)	To evaluate the associations of physical activity with periodontitis and investigate if the relationship between physical activity and inflammatory mediators differs between periodontitis cases and non-cases.	10.1111/j.1600-051X.2009.01394.x
Staufenbiel,et al. (2023)	Evaluate the association of specific dietary ingredients and physical activity on local and systemic inflammatory markers in experimentally induced gingivitis	10.3390/nu15153344
Tada, et al. (2003)	To evaluate the relationship between oral health and physical activities	10.1016/S0167-4943(03)00026-8
Torrejon-Moya, et al. (2021)	To investigate if lifestyle habits affect the oral health status of patients who attend the Dental Hospital of the University of Barcelona	10.3390/ijerph18147488
Tsai,et al. (2024)	Evaluate the association between guideline-based levels of physical activity and periodontitis in young adults	10.1016/j.jds.2023.05.036

Xu, et al. (2021)	To examine the associations between health behaviors and oral health and investigate the potential moderating role of education and living arrangements in such relationships.	10.3390/ijerph18147285
Xu,et al. (2024)	Investigate the association between periodontitis and different combinations of healthy lifestyle	10.1186/s12903-024-03937-z
Yoshimoto, et al. (2022)	To evaluate the effect of walking training "Interval Walking Training" on oral health status.	10.3390/ijerph192114465
