# Supplementary material for: Twenty-first century knowledge mapping on oral diseases and physical activity/exercise, trends, gaps, and future perspectives: a bibliometric review
Source: Front Sports Act Living. 2024 Aug 7;6:1410923. doi: 10.3389/fspor.2024.1410923 (PMC11335734; doi:10.3389/fspor.2024.1410923)
Supplement: Supplementary file 4 [file Datasheet4.pdf]

Supplementary Material

Supplementary Table 4. Index of studies that evaluated the impact of oral diseases on sports performance and physical fitness

Author (year)	*Main Objective*	*DOI*
Angelozzi, et al. (2008)	To examine the relationship between dental occlusion, physical performance and half squat posture, respectively.	Medicina dello Sport 2008 June;61(2):147-57
Battaglia, et al. (2016)	Investigate the influence of increased DVO on cervical spine mobility in individuals with or without malocclusion, analyzing both sports and sedentary subjects.	10.19193/0393-6384_2016_5_135
D'Ercole, et al. (2013)	To evaluate the oral health status in young swimmers, assessing salivary cariogenic bacteria and the concentration of S-IgA before and after training sessions.	PMID: 23830404
Eremenko, et al. (2016)	Investigate a relationship between periodontitis and muscle strength	10.1111/jcpe.12531
Ferrario, et al. (2001)	To assess the relationship between occlusal conditions and neuromuscular performance	10.1046/j.1365-2842.2001.00749.x
Gallagher, et al. (2018)	To assess dental caries, erosive tooth wear, periodontal health, self-reported oral health problems, and performance impacts in aUK elite athletes from different sports	10.1111/cdoe.12392
Gallagher, et al. (2020)	To assess the feasibility and effectiveness of simple preventive interventions to improve oral health knowledge, promote oral health behavior, and minimize performance impacts among elite athletes.	10.1136/bmjsem-2020-000759
Gaszynska, et al. (2014)	To evaluate the association between masseter muscle tension, dental status, and parameters of physical fitness.	10.2147/CIA.S66672
Harel, et al. (2021)	To examine the relationship between physical performance and the geriatric masticatory coefficient in patients who underwent both a comprehensive gerontological assessment	10.1016/j.archger.2021.104421

Holtfreter, et al. (2021)	To investigate the association between clinically assessed periodontitis and cardiopulmonary exercise testing	10.1177/0022034521995428
Hong, et al. (2023)	Investigate associations between probability of osteoporotic fractures and oral health in the elderly.	10.1038/s41598-023-28650-9
Hoppe, et al. (2017)	To assess the relationship between chronic oral inflammatory burden, which includes both periodontal and endodontic disease load, and physical fitness.	10.1111/iej.12686
Iinuma, et al. (2012)	To evaluate the relationship between mastication and physical performance in long-lived elderly individuals.	10.1111/j.1532-5415.2011.03780.x
Iwao, et al. (2019)	To assess the effectiveness of a comprehensive long-term care prevention program, including physical exercise, oral health education, and nutritional guidance, for elderly individuals living in the community.	10.1002/cre2.226
Karjalainen, et al. (2002)	Investigate the oral health status and the need for dental treatment in institutionalized patients with intellectual disabilities, while associating it with the level of physical activity	10.1080/000163502753472005
Kim,et al. (2023)	Investigate the relationship between the number of remaining teeth and grip strength	10.3390/medicina59081373
Klett (2019)	To test the hypothesis that A dysfunction of the temporomandibular joint may worsen the golf swing.	10.1007/s00337-019-0531-9
Meisel, et al. (2023)	Identify the interaction between masticatory muscles, teeth and general muscle fitness and their contribution to body adiposity.	10.1007/s10266-023-00785-1
Murotani, et al. (2021)	Investigate which oral functions are associated with muscle strength (grip strength), physical performance (walking speed) or both.	10.3390/ijerph182413199

Musacchio, et al. (2021)	To investigate the association of the number of remaining teeth with physical functioning and disability in aging Italian men and women	10.1371/journal.pone.0255741
Nagaeva, et al. (2023)	Evaluate the variability of habitual motor activity in young individuals with connective tissue dysplasia and periodontitis	10.14529/hsm230119
Needleman, et al. (2013)	To evaluate the oral health of athletes participating in the London 2012 Games, including oral health determinants and their effect on the well-being, training, and performance	10.1136/bjsports-2013-092891
Oliveira, et al. (2015)	Investigate whether periodontal disease is an indicator of risk for poor physical fitness.	10.1902/jop.2014.140270
Roth, et al. (2024)	Assess the association between self-reported periodontal disease and gingival bleeding as predictors of handgrip strength	10.1007/s00784-024-05507-7
Saito, et al. (2023)	Examine the association between locomotive syndrome and oral health status, such as the number of teeth and chewing function, in an adult population	10.1186/s12877-023-04572-z
Sasajima, et al. (2021)	To evaluate the effects of oral function training and oral health status on improvements in physical performance in dependent older adults.	10.3390/ijerph182111348
Solleveld, et al. (2018)	Explore associations of poor oral health and of use of a Fixed orthodontic appliances with injury frequency and postural stability.	10.1186/s13102-018-0105-5
Solleveld, et al. (2022)	To evaluate the relationships between masticatory muscles asymmetry, oral health, postural control, and the prevalence of leg injuries	10.2478/hukin-2022-0086
Takata, et al. (2004)	To assess the relationship between masticatory ability, number of teeth, and measures of physical fitness	10.1046/j.1354-523X.2003.00972.x

Thai, et al. (2014)	To evaluate the association between periodontal infection and cardiorespiratory fitness levels among a population sample of younger adults.	10.1371/journal.pone.0092441
Von Held, et al. (2021)	To assess the impact of dental caries on OHRQoL in Para athletes and to evaluate potential biomarkers for OHRQoL in Para athletes.	10.1111/scd.12627
