# Supplementary material for: Twenty-first century knowledge mapping on oral diseases and physical activity/exercise, trends, gaps, and future perspectives: a bibliometric review
Source: Front Sports Act Living. 2024 Aug 7;6:1410923. doi: 10.3389/fspor.2024.1410923 (PMC11335734; doi:10.3389/fspor.2024.1410923)
Supplement: Supplementary file 5 [file Datasheet5.pdf]

Supplementary Material

Supplementary Table 5. Index of studies that associated Oral health status, physical exercise and systemic alterations

Author (year)	***Main Objective***	***DOI***
Alkan, et al. (2020)	Investigate the effects of regular physical exercise in obese women with periodontal disease, using samples of serum, saliva, and gingival crevicular fluid.	10.4103/njcp.njcp_627_19
Aswathikutty, A, et al. (2017)	Explore the interrelationship of obesity and physical activity at age 11-12 years with TDI at age 15-16 years among schoolchildren from East London.	10.1111/edt.12318
Chen,et al. (2023)	Explore the link between periodontitis and uncontrolled hypertension, investigating if inflammation markers like white blood cell (WBC) or neutrophil counts mediate this connection	10.1186/s12903-023-03680-x
Ferrillo,et al. (2023)	Investigate systemic and oral health parameters in eutrophic, overweight, and obese adolescents	10.3390/jpm13071073
Hasan, SMM, et al. (2021)	To evaluate the relationship between periodontal disease and the diabetes patients' self-care practices and oral hygiene-related knowledge, attitudes and behaviours	10.1371/journal.pone.0249011
Notohartojo, IT, et al. (2019)	To determine the relationship between hypertension, physical activity and brushing technique with periodontal disease in Indonesia.	10.15562/bmj.v8i1.1324
Portes, J, et al. (2023)	To evaluate the prevalence of diabetes and prediabetes in the elderly patients who attended a dental clinic and to find common risk factors.	10.1016/j.jdent.2023.104480
Schulze, A, et al. (2023)	Investigate the effects of exercise training on periodontal inflammation specifically in diabetic patients.	10.23736/S0022-4707.22.14197-6
Shimazaki, Y, et al. (2010)	Investigate the relationship between obesity, physical fitness, and periodontitis.	10.1902/jop.2010.100017

Uchida, F, et al. (2021)	To analyze the oral microflora and compare the metabolic activities of the microflora of obese patients with NAFLD and periodontal disease before and after a 12-week exercise regimen.	10.3390/ijerph18073470
Vivekananda, L, et al. (2019)	To evaluate the impact of weight reduction on the attenuation of obesity-related periodontal inflammation.	10.4103/jispcd.JISPCD_447_18
Wernicke, K, et al. (2021)	Investigate the effect of physical activity on periodontal health and HbA1c levels in patients with type 2 diabetes mellitus over a period of 6 months.	10.1007/s00784-021-03908-6
